# DNA methylation profiling allows for characterization of atrial and ventricular cardiac tissues and hiPSC-CMs

**DOI:** 10.1186/s13148-019-0679-0

**Published:** 2019-06-11

**Authors:** Kirstin Hoff, Marta Lemme, Anne-Karin Kahlert, Kerstin Runde, Enrique Audain, Dorit Schuster, Jens Scheewe, Tim Attmann, Thomas Pickardt, Almuth Caliebe, Reiner Siebert, Hans-Heiner Kramer, Hendrik Milting, Arne Hansen, Ole Ammerpohl, Marc-Phillip Hitz

**Affiliations:** 1 Department of Congenital Heart Disease and Pediatric Cardiology, University Hospital Schleswig-Holstein, Campus Kiel, Kiel, Germany; 2 DZHK (German Centre for Cardiovascular Research), partner site Hamburg/Kiel/Lübeck, Hamburg, Germany; 3 Department of Experimental Pharmacology and Toxicology, University Medical Center Hamburg-Eppendorf, Hamburg, Germany; 4 Institute for Clinical Genetics, Carl Gustav Carus Faculty of Medicine, Dresden, Germany; 5 Institute of Human Genetics, Christian-Albrechts-University Kiel & University Hospital Schleswig-Holstein, Campus Kiel, Kiel, Germany; 6 National Register for Congenital Heart Defects, DZHK (German Centre for Cardiovascular Research), Berlin, Germany; 7 Competence Network for Congenital Heart Defects, DZHK (German Centre for Cardiovascular Research), Berlin, Germany; 8 Institute of Human Genetics, University Hospital Ulm, Ulm, Germany; 9 Erich and Hanna Klessmann Institute for Cardiovascular Research & Development (EHKI), Heart and Diabetes Center NRW, Ruhr University Bochum, Bad Oeynhausen, Germany; 10 Wellcome Trust Sanger Institute, Cambridge, UK

**Keywords:** DNA methylation, Cardiac tissue-specific DNA methylation, Human induced pluripotent stem cell derived cardiomyocytes (hiPSC-CM), Engineered heart tissue (EHT), Bisulfite pyrosequencing, 450K array

## Abstract

**Background:**

Cardiac disease modelling using human-induced pluripotent stem cell-derived cardiomyocytes (hiPSC-CM) requires thorough insight into cardiac cell type differentiation processes. However, current methods to discriminate different cardiac cell types are mostly time-consuming, are costly and often provide imprecise phenotypic evaluation. DNA methylation plays a critical role during early heart development and cardiac cellular specification. We therefore investigated the DNA methylation pattern in different cardiac tissues to identify CpG loci for further cardiac cell type characterization.

**Results:**

An array-based genome-wide DNA methylation analysis using Illumina Infinium HumanMethylation450 BeadChips led to the identification of 168 differentially methylated CpG loci in atrial and ventricular human heart tissue samples (*n* = 49) from different patients with congenital heart defects (CHD). Systematic evaluation of atrial-ventricular DNA methylation pattern in cardiac tissues in an independent sample cohort of non-failing donor hearts and cardiac patients using bisulfite pyrosequencing helped us to define a subset of 16 differentially methylated CpG loci enabling precise characterization of human atrial and ventricular cardiac tissue samples. This defined set of reproducible cardiac tissue-specific DNA methylation sites allowed us to consistently detect the cellular identity of hiPSC-CM subtypes.

**Conclusion:**

Testing DNA methylation of only a small set of defined CpG sites thus makes it possible to distinguish atrial and ventricular cardiac tissues and cardiac atrial and ventricular subtypes of hiPSC-CMs. This method represents a rapid and reliable system for phenotypic characterization of in vitro-generated cardiomyocytes and opens new opportunities for cardiovascular research and patient-specific therapy.

**Electronic supplementary material:**

The online version of this article (10.1186/s13148-019-0679-0) contains supplementary material, which is available to authorized users.

## Background

DNA methylation plays a critical role during early mammalian development [1, 2] by regulating transcriptional processes [3, 4]. Studies have highlighted the impact of distinct DNA methylation patterns during cell type specification and organ maturation [5–7] of highly specialized organs, such as the human heart [8]. The fine-tuned spatial and temporal DNA methylation process is not only relevant to early heart development [9–11], but it also influences cardiac disease and its progression [12] [13] by impacting, among others, cardiomyocyte maturation [14]. However, the epigenetic process shaping different cardiac cell types (e.g. pacemaker, atrial and ventricular cells) remains poorly understood.

Thorough insight into cardiac maturation and the molecular mechanisms driving cell type developmental and differentiation processes is essential to understand the different aspects of heart disease. Currently, the majority of the available cell culture disease models only unreliably mirror the in vivo condition. The introduction of human-induced pluripotent stem cell (hiPSC) technology [15] [16], including hiPSC cardiomyocytes (hiPSC-CMs) [17], opens new opportunities for cardiovascular research, drug-screening and patient-specific therapy [18]. hiPSC-CMs and their technical improvements, such as 3D force-generating engineered heart tissues (EHTs) [19] [20] [21], have dramatically increased our ability to reproducibly model different types of cardiac disease [22, 23]. Nevertheless, the precise molecular characterization of these cell cultures still remains challenging. Up to now, the distinction of different cardiac cell types was accomplished by analysing transcript levels of cardiac surface marker genes, histological stainings or electrophysiological techniques [23–28]. However, many of these methods are costly and laborious proceedings.

**Fig. 1:**
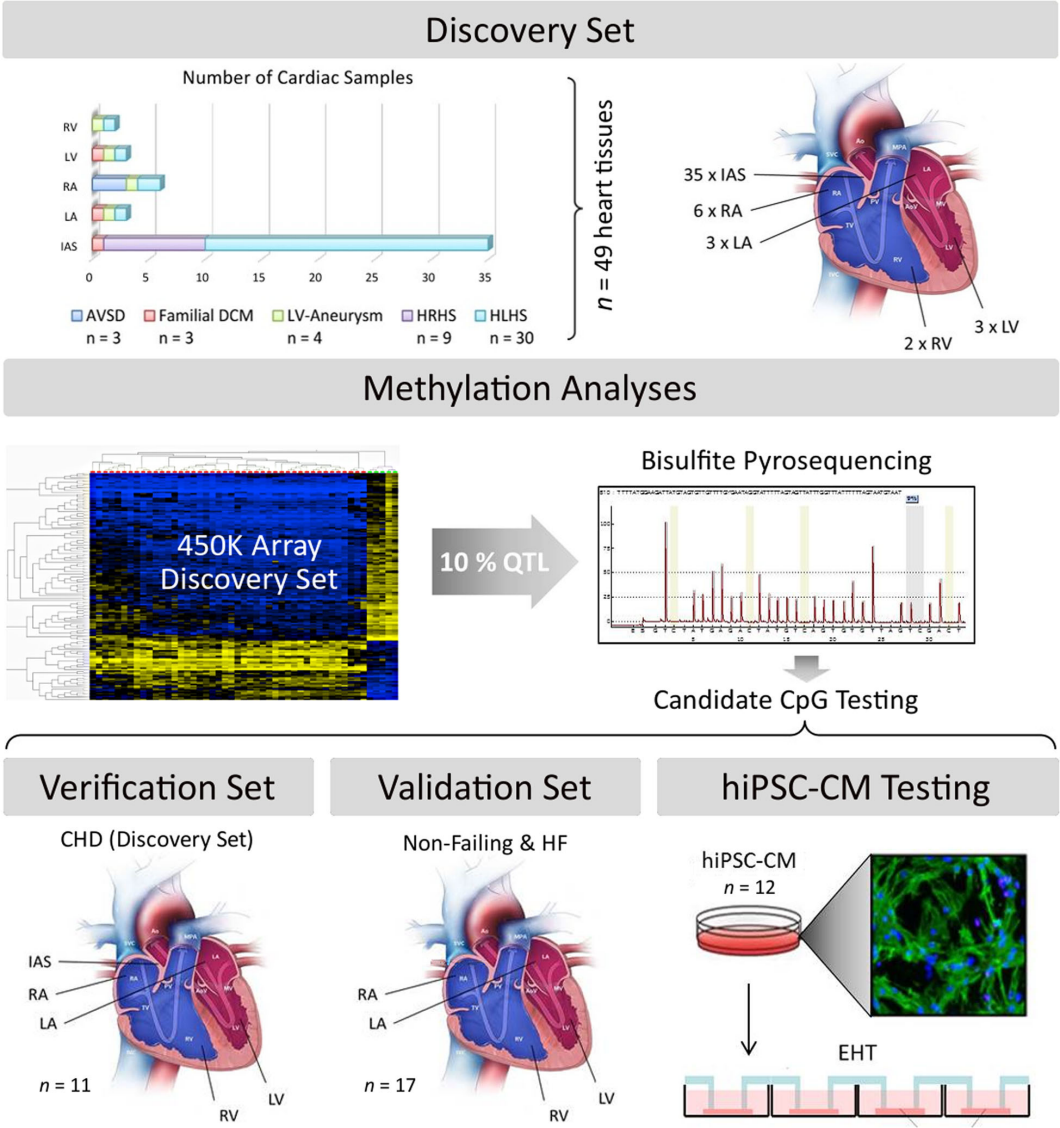
Study design. A discovery set comprising 49 human cardiac tissue samples from paediatric patients with different congenital heart diseases (CHD) was subjected to DNA methylation analysis using Illumina Infinium HumanMethylation450 BeadChips (‘450K arrays’). Having identified significant differential methylation patterns in atrial (left and right atrium (LA, RA), interatrial septum (IAS)) and ventricular (left and right ventricle (LV, RV)) tissues, the 10% quantile of CpG loci with greatest delta *β*-values among the differentially methylated CpG loci (16 loci) was selected to further evaluate their reproducibility using bisulfite pyrosequencing. As verification set, 11 heart tissue samples from the initial analysis were subjected to bisulfite pyrosequencing, followed by the analysis of 13 non-failing heart tissue samples from non-transplantable donor hearts as well as 4 heart tissue samples from adult patients with heart failure (HF) (validation set *n* = 17). To further distinguish the cellular identity of in vitro-generated human-induced pluripotent stem cell-derived cardiomyocytes (hiPSC-CMs), batches of atrial and ventricular hiPSC-CMs (*n* = 12) from 2D monolayers and 3D-engineered heart tissues (EHT) were subjected to bisulfite pyrosequencing. For detailed sample information, see Additional file 14: Table S1. Credits: images partly adapted from Centers for Disease Control and Prevention, National Center on Birth Defects and Developmental Disabilities [29] and Mannhardt *et al*., 2016 [26]

Here, we investigated array-based genome-wide DNA methylation patterns and regional methylation profiles of different human cardiac tissues from non-transplantable (non-failing) donor hearts and cardiac patients, as well as hiPSC-CMs (study design Fig. 1), to determine whether they can be distinguished by their DNA methylation. First, we systematically applied genome-wide DNA methylation profiling of cardiac tissue samples (*n* = 49) from patients with different congenital heart diseases (CHD) using Illumina Infinium HumanMethylation450 BeadChips (‘450K arrays’). This enabled us to identify significant differential DNA methylation patterns clearly discriminating human atrial and ventricular cardiac tissue samples. Validation and verification of the observed atrial-ventricular regional DNA methylation pattern was done using bisulfite pyrosequencing in an independent cardiac sample cohort (*n* = 17) consisting of non-failing donor hearts and samples from different cardiac patients, as well as by analysing different hiPSC-CM subtypes (*n* = 12) to clarify their cellular identity.

We provide evidence for a distinct DNA methylation profile related to different cardiac tissue types. A key finding of our study is the discovery of a defined set of CpG dinucleotides (CpG loci) to reproducibly distinguish atrial and ventricular subtypes of hiPSC-CMs by their significant differential DNA methylation that further confirmed their previously measured phenotypic expression profiles. Using these techniques, we established a rapid and cost-effective method allowing the characterization of atrial and ventricular hiPSC-CMs for further use as cardiac disease-modelling systems.

## Results

### Differential DNA methylation pattern between atrial and ventricular human cardiac tissues

**Fig. 2:**
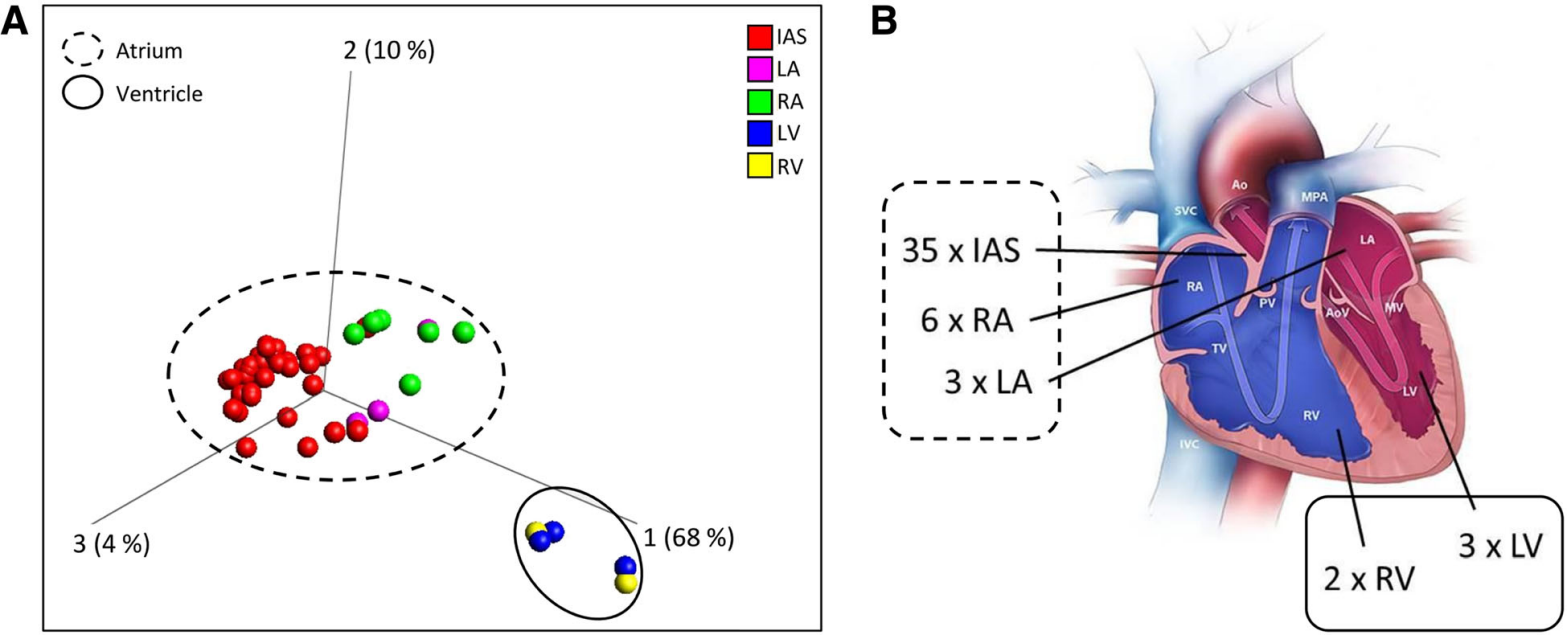
Principal component analysis of *β*-values at differentially methylated CpG loci from 49 cardiac tissue samples. **a** Principal component analysis based on *β*-values of differentially methylated 271 CpG loci (*q* ≤ 1 × 10^−6^ (*σ*/*σ*_max_ > 0.4), ANOVA) of 49 different cardiac tissue samples (IAS, LA, RA, LV, RV) that were subjected to 450K array analysis. Subgroups of atrial and ventricular samples are marked as dashed and solid edging, respectively **b**. IAS: red spheres, LA: pink spheres, RA: green spheres, LV: blue spheres, RV: yellow spheres. Credits: Centers for Disease Control and Prevention, National Center on Birth Defects and Developmental Disabilities [29]

Using the 450K array platform [30], assessing the methylation status of more than 485,000 loci, we analysed different heart tissue samples from patients with congenital heart defects (CHD) (for detailed information, see Additional file 14: Table S1). Subsequent to data preprocessing and quality filtering, the final data matrix comprised *β*-values (methylation levels) across 448,814 loci in 49 cardiac tissue samples for further statistical analyses. No differences in DNA methylation could be observed between different types of cardiac disease (Additional file 1: Figure S1) in unsupervised PCA. No batch effects due to sample distribution on the array occurred (Additional file 2: Figure S2). Biological replicates showed high *β*-value correlation with *R*^2^ > 0.99 (*p* < 2.2 × 10^−16^) (Additional file 3: Figure S3) across the 448,814 loci. ANOVA statistical analysis of different cardiac tissue types (IAS, RA, LA, RV, LV) among the 49 samples revealed 271 differentially methylated loci with a FDR ≤ 1 × 10^−6^ (*σ*/*σ*_max_ > 0.4), depicted as principal component analysis (PCA) in Fig. 2a (unsupervised PCA, see Additional file 4: Figure S4).

**Fig. 3:**
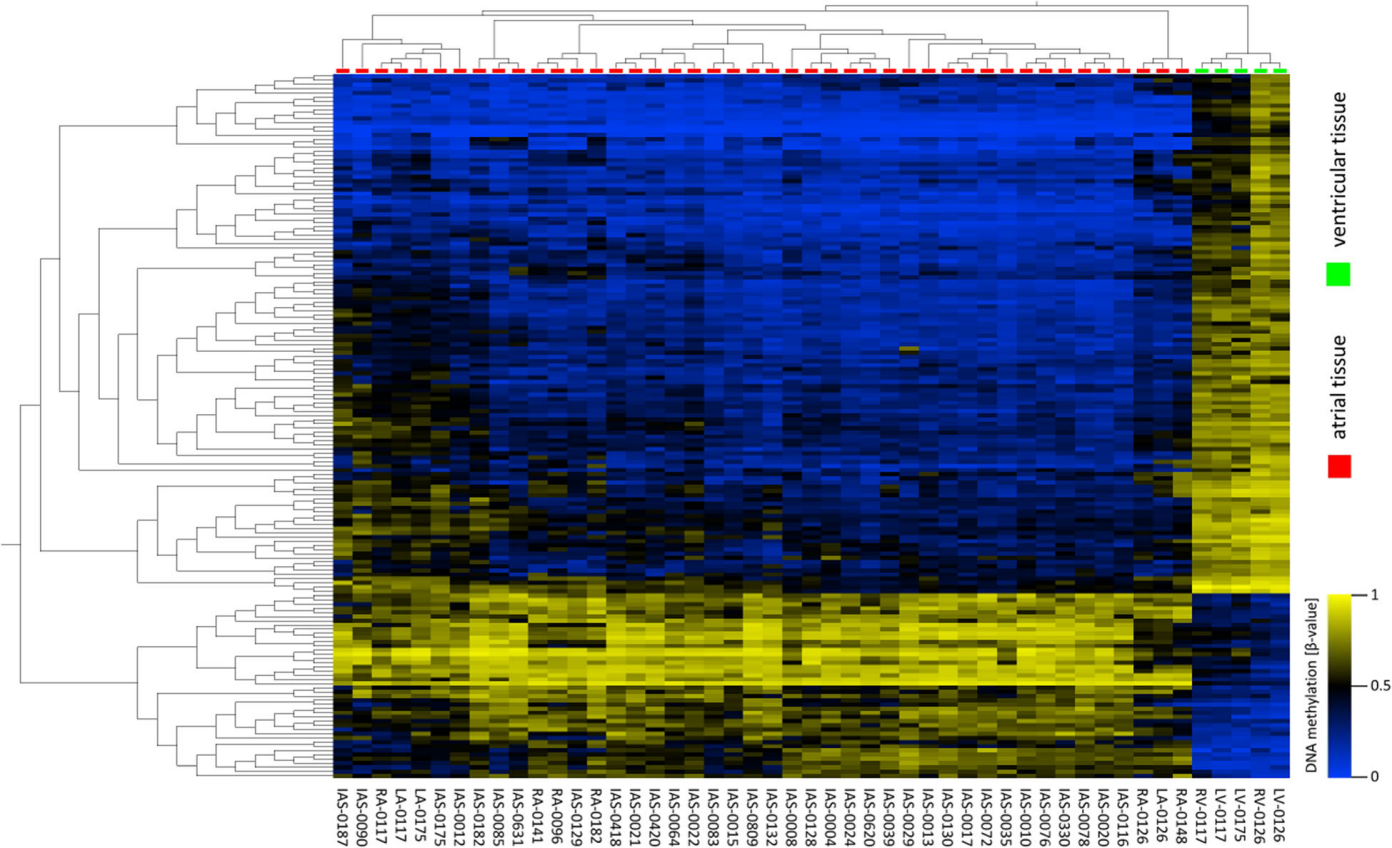
Hierarchical clustering (*t* test) of differentially methylated CpG loci in 49 cardiac tissue samples. One hundred and sixty-eight differentially methylated CpG loci (*q* ≤ 1 × 10^−6^ (*σ*/*σ*_max_ > 0.4) *t* test) between 44 atrial (columns marked in red) and 5 ventricular heart tissue samples (columns marked in green) are depicted as hierarchical clustering analysis. Each column represents one sample (sample names and tissue types below), in case of sample 0126 and 0117 biological replicates are presented as mean values (for detailed information, see Additional file 14: Table S1). Each horizontal line represents the methylation levels of a given CpG loci across samples. Methylation levels are expressed as *β*-values from 0 to 1 (blue and yellow, unmethylated and completely methylated, respectively). All CpG loci show a difference of at least Δ*β* ≥ 0.3 (up to Δ*β* = 0.6) in their DNA methylation values between atrial and ventricular samples. No normalization was applied to colour code of the heat map, real *β*-values are shown

A *t* test was applied to identify differentially methylated CpG loci between atrial (defined as LA, RA, IAS) and ventricular (LV, RV) heart tissues, resulting in 168 significantly differentially methylated CpG loci (FDR of < 1 × 10^−6^ (σ/σ_max_ > 0.4), Additional file 15: Table S2), as shown in Fig. 3. Atrial tissues revealed mostly a hypomethylation while ventricular tissues showed a hypermethylation at 74% of CpG loci. High delta *β*-values (absolute differences of *β*-values between two sample cohorts) were observed, ranging from Δ*β* = 0.3 to Δ*β* = 0.6 with 54% of loci having a Δ*β* ≥ 0.4. Fifty percent of CpGs (84/168) were overlapping with predicted enhancer elements (OR = 3.6, *p* = 5.9 × 10^−16^), and 14% (24/168) showed overlap with DNAse I hypersensitivity sites determined by ENCODE Project Consortium [31] (Additional file 5: Figure S5). Associations to UCSC CpG islands occurred at 13% of CpGs (22/168), while shores and shelfs of CpG islands showed an overlap of 29% (48/168) and 7% (12/168), respectively. Associations to UCSC gene regions showed the highest proportion in gene bodies (30%, 51/168), regions up to 200 bp upstream of transcriptional start sites (TSS200) were overlapping with 8% of CpGs (13/168) and TSS1500, 5′UTR, 3′UTR and promotors showed an overlap of 7% (11/168), 11% (19/168), 4% (6/168) and 4% (6/168), respectively (Additional file 5: Figure S5 and detailed information in Additional file 15: Table S2). Besides CpG island shores, which showed a higher proportion in ventricular-hypo/atrial-hypermethylated CpGs as compared to ventricular-hyper/atrial-hypomethylated CpGs, all other regulatory features or gene regions did not show any difference between heart tissues.

Transcription factor binding sites (TFBS) subject to differential DNA methylation among the 168 CpGs were analysed by comparing the CpG loci with TF ChIP-seq performed by ENCODE (Factorbook Motifs [32]). Fifty-four percent (90/168) of differentially methylated CpGs were associated to different TFBSs in various cell lines (detailed information in Additional file 15: Table S2). The ten most common TFs showing TFBSs among the 168 CpGs were POLR2A, EZH2, EP300, GATA2, FOS, RUNX3, CEBPB, TCF7L2, MAX and TEAD4 (Additional file 6: Figure S6). There were no associations to any cardiac cell lines (AoAF, HCFaa, HCM, HPAF), nor to cardiac TFs (MEF2A, MEF2C, SRF). Over-representation analysis by Reactome Pathways Tool [33] linking genes with differentially methylated CpGs and associations to TFBSs resulted in the following significant (*q* < 0.01) pathway with ≥ 3 genes: ‘Developmental Biology’ (R-HSA-1266738, *q* = 6.3 × 10^−3^) (Additional file 15: Table S2). With two genes per pathway, ‘Signaling by FGFR in disease’ (R-HSA-1226099 (*STAT3*, *FGFR2*), *q* = 4.6 × 10^−5^) and ‘Activation of HOX genes during differentiation’ (R-HSA-5619507 (*HOXA3*, *HOXB3*), *q* = 7.4 × 10^−4^) were represented.

In order to investigate possible similarities in DNA methylation patterns potentially involved in developmental and differentiation processes, MBD-Seq data of DNA methylation in heart tissues of mice [34] was compared to the 450K data of the present study. All in all, 4 CpG loci of the 168 differentially methylated CpG loci showed an overlap to MBD-Seq reads of Sim et al. (2014) [34]. The ± 60 bp flanking array sequences of three CpG loci—cg18177275, cg12924936 and cg13706058—overlapped with different query sequences of the MBD-Seq analysis, but the specific CpG loci were not targeted (Additional file 15: Table S2). Only one CpG locus, cg04115185, showed overlap to one MBD-Seq read of mouse P1 data (92% homology).

The 168 differentially methylated loci could be annotated to 78 RefSeq genes. Overlapping CpG loci of these 78 genes with predicted human heart enhancers from a study of Dickel et al. [35] resulted in 24 CpG loci (24/168 loci, OR = 0.96, *p* = 0.92) (for details, see Additional file 15: Table S2). Multiple CpG loci overlapping with heart enhancers could be identified in three genes: *IRX4* (3 loci), *NAV1* (2 loci) and *TBX5* (2 loci), with confidence scores of each predicted enhancer [35] of 0.402, 0.539 and 0.372, respectively. Gene ontology analysis using PANTHER14.0 [36] showed significant enrichment for genes involved in different developmental GO biological processes. Among the top ten with highest fold enrichment (Additional file 15: Table S2) ‘cardiovascular system development’ (GO:0072358, *q* = 1.96 × 10^−2^), ‘animal organ morphogenesis’ (GO:0009887, *q* = 2.02 × 10^−4^), ‘embryonic morphogenesis’ (GO:0048598, *q* = 2.95 × 10^−2^) and ‘positive regulation of cell differentiation’ (GO:0045597, *q* = 2.94 × 10^−2^) were observed.

Furthermore, expression of DNA methyltransferases was tested to analyse if atrial and ventricular cardiac tissues showed any differences in expression levels. Given the low residual tissue amounts, atrial and ventricular cardiac tissue from only one patient (patient 0126) was used for expression analysis of *DNMT1* and *DNMT3A*. While the expression of *DNMT1* showed no significant differences between atrial and ventricular tissues, *DNMT3A* revealed significantly higher expression in atrial tissue compared to ventricular tissue (Additional file 7: Figure S7).

### Identification of candidate CpG loci for distinction of atrial and ventricular cardiac tissues

**Fig. 4:**
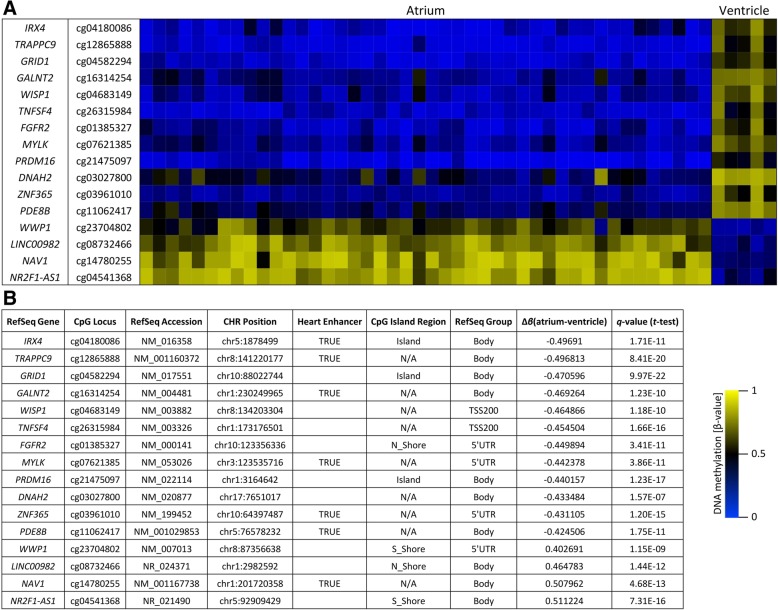
Candidate CpG loci with differential atrial-ventricular methylation pattern. Identified CpG loci (16 loci) and associated genes with greatest DNA methylation value differences (Δ*β*-value) between atrial and ventricular samples based on the *t* test (*q* ≤ 1 × 10^−6^, *σ*/*σ*_max_ > 0.4) comparing 450K array data of 44 atrial and 5 ventricular heart tissue samples (**a**). All CpG loci show a difference of at least Δ*β* ≥ 0.4 in their DNA methylation values (see column ‘Δ*β*(atrium-ventricle)’) between atrial and ventricular samples (**b**). Additional information regarding chromosomal position (CHR), CpG island regions (N_Shore, 0–2 kb upstream (5′); S_Shore, 0–2 kb downstream (3′) of island) and RefSeq Group gene position annotation (TSS200, 0–200 bases upstream of the transcriptional start site; body, intragenic localization of the CpG site) are given according to Illumina’s 450K array classifications and UCSC classifications [37]. Heart enhancer, overlap of CpG locus with predicted human heart enhancers identified by a study of Dickel et al. [35] are marked as ‘true’ (for details, see Additional file 15: Table S2). These 16 CpG loci were selected as candidate CpG loci to test further sample cohorts using bisulfite pyrosequencing

Selecting the upper 10% quantile (based on Δ*β*-value) of differentially methylated CpG loci between atrial and ventricular heart tissues (*q* ≤ 1 × 10^−6^, *σ*/*σ*_max_ > 0.4), 12 CpG loci with atrial hypomethylation and 4 CpG loci with atrial hypermethylation were applied as candidate CpG loci for further analysis (Fig. 4). Additional *Welch two*-*sample t* test comparing the *β*-values from atrial and ventricular samples of the 16 candidate CpG loci confirmed highly significant DNA methylation differences (Additional file 8: Figure S8) with a mean delta *β*-value of Δ*β* = 0.46. In total, these 16 candidate CpG loci comprised 7 loci with predicted human heart enhancers from a study of Dickel et al. (2016) [35] (for details, see Additional file 15: Table S2). Ten CpG loci were located intragenic, while six CpG loci were associated with the 5′-flanking region or the transcriptional start site (Fig. 4b). The 16 candidate CpG loci were mainly non-island CpGs. Three loci were associated to CpG islands (*IRX4*, *GRID1*, *PRDM16*).

**Fig. 5:**
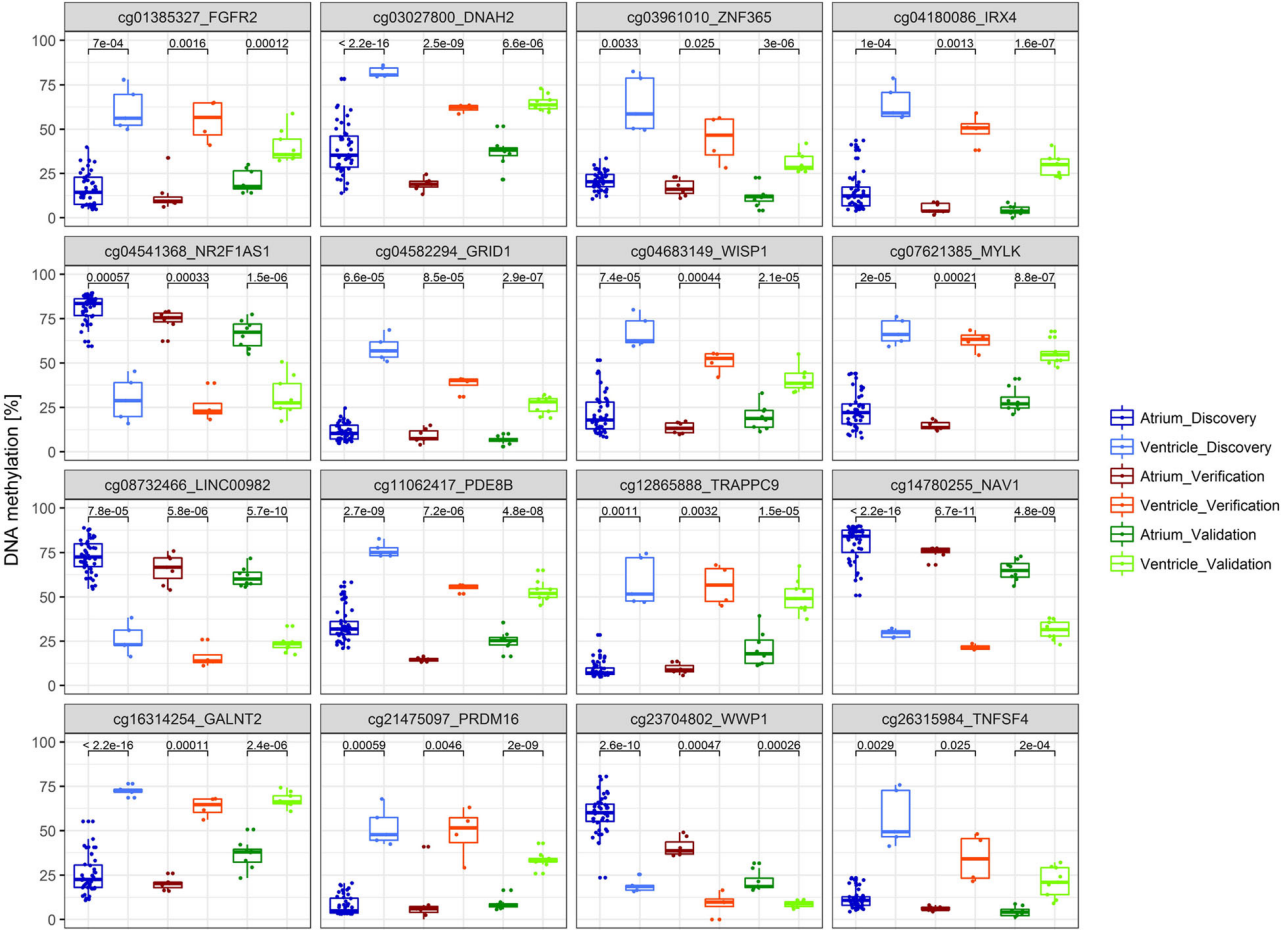
Bisulfite pyrosequencing analysis of verification and validation set. Bisulfite pyrosequencing of 16 candidate CpG loci in atrial and ventricular cardiac tissue samples (detailed sample information, see Additional file 14: Table S1). The DNA methylation values (percentage) of three analysis sets are depicted: (1) initial discovery set (450K array analysis of atrial (*n* = 44, coloured dark blue) and ventricular (*n* = 5, coloured light blue) cardiac tissues from patients with CHDs), (2) verification set (atrial samples (*n* = 7, coloured dark red) and ventricular samples (*n* = 4, coloured orange) from the initially analysed sample cohort of the discovery set) and (3) validation set (four heart tissue samples (1x LA and 3x RA tissue) from adult patients with heart failure and 13 non-failing heart tissue samples (*n* = 4 atrial and *n* = 9 ventricular samples; coloured dark green and light green, respectively). All 16 candidate CpG loci show significant (*p* < 0.05) DNA methylation differences in atrial compared to ventricular cardiac tissue samples. Data is presented as standard box-and-whiskers plots (whiskers, 5th—95th percentile)

To verify the atrial-ventricular DNA methylation (AVM) pattern, seven atrial samples (IAS, LA, RA from hypoplastic left heart syndrome (HLHS) or LV aneurysm patients, respectively) and four ventricular samples (LV, RV from HLHS or LV aneurysm patients, respectively) from the initially analysed sample cohort were subjected to bisulfite pyrosequencing (detailed sample information, see Additional file 14: Table S1). DNA methylation values measured by 450K array (discovery set) and those measured by bisulfite pyrosequencing (verification set) showed a high correlation with a mean *R*^2^ = 0.97 (*R*^2^ ranging from > 0.99 to > 0.89, and *p* values ranging from *p* = 6.9 × 10^−10^ to *p* = 0.00026) among the 16 CpG loci (for detailed information, see Additional file 9: Figure S9, Additional file 10: Figure S10, Additional file 11: Figure S11 and Additional file 12: Figure S12). As depicted in Fig. 5, the AVM pattern could be verified at all tested 16 CpG loci showing highly significant DNA methylation differences between atrial and ventricular heart tissues (mean Δ%-methylation = 40.8%) with similar DNA methylation pattern as in the primary 450K array-based DNA methylation analysis.

To assess the biological significance of the detected AVM pattern and to rule out cardiac disease-related effects, 13 non-failing heart tissue samples (four atrial and nine ventricular samples) were analysed as an independent validation set. Furthermore, four heart tissue samples (LA and RA tissue) from adult patients with heart failure were included (detailed sample information, see Additional file 14: Table S1). By DNA methylation analysis of these 16 CpG loci, we could consistently discriminate between atrial and ventricular tissues (mean Δ%-methylation = 25%) in this independent validation set (Fig. 5). Although variations in absolute methylation values occurred between the different sample cohorts, all 16 CpG loci showed (highly) significant DNA methylation differences. We could demonstrate a 100% predictability of heart tissue type classification in this independent sample cohort. The DNA methylation values of samples analysed by bisulfite pyrosequencing are listed in Additional file 18: Table S5 (*p* values Additional file 19: Table S6).

### Specification of hiPSC cardiomyocyte subtypes

**Fig. 6:**
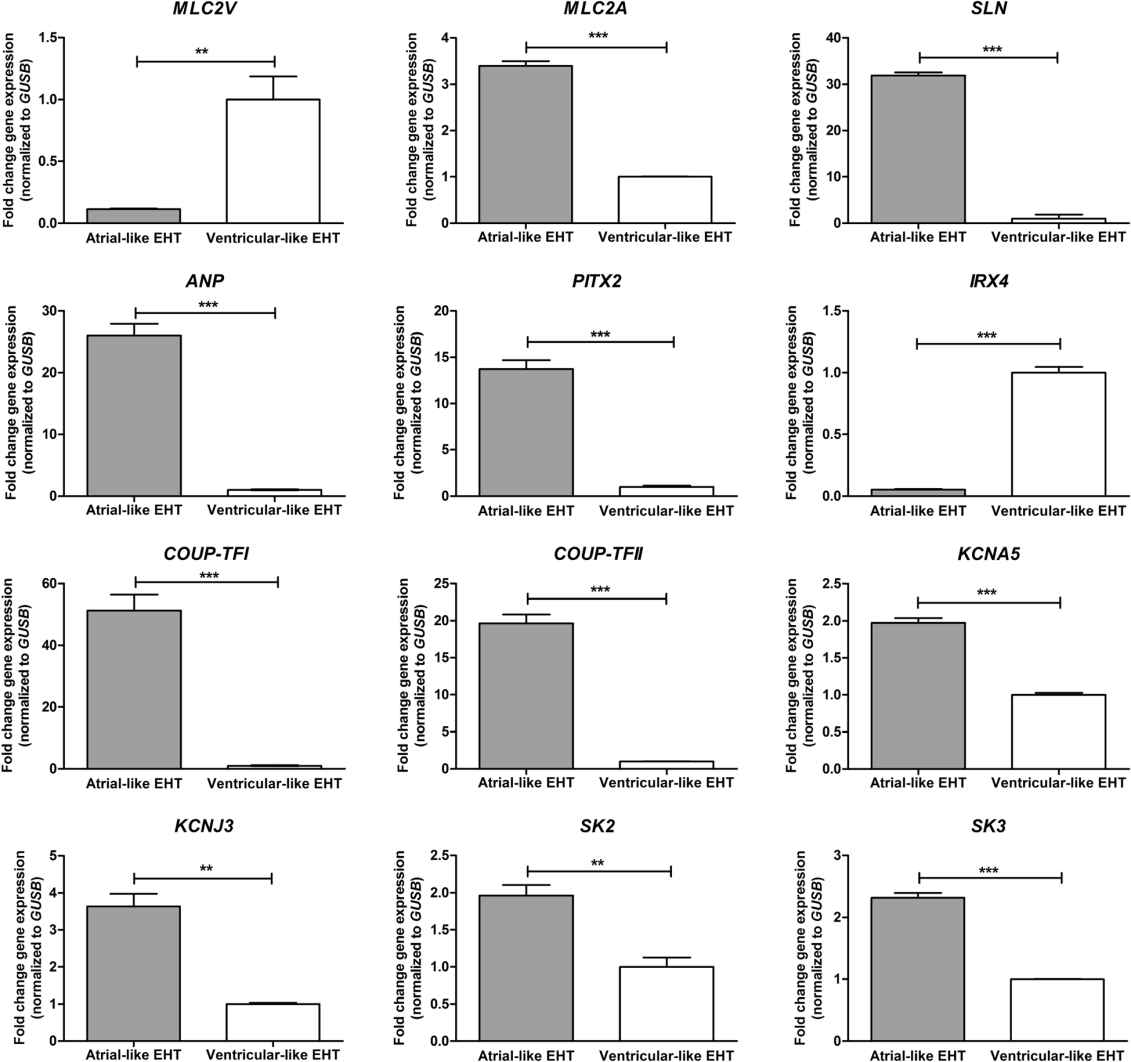
mRNA expression of selected genes in atrial-like and ventricular-like hiPSC-CM EHTs. qPCR experiments were performed on atrial and ventricular-like hiPSC-CMs from three independent hiPSC-CM EHT generations, each as duplicates (*n* = 6). Expression data was normalized to *GUSB* housekeeping gene and compared to atrial or ventricular-like hiPSC-CM EHT expression (∆∆ CT method), *p* < 0.05 (Student’s *t* test), bars show mean ± SEM

To characterize cultivated atrial and ventricular-like subtypes of hiPSC-CMs, mRNA expression analyses of atrial and ventricular-specific genes were performed using quantitative real-time PCR (qPCR). qPCR data of 12 genes of hiPSC-CM EHTs is presented in Fig. 6. Also, expression analysis of these 12 genes of atrial and ventricular-like hiPSC-CM 2D monolayers has been performed using the same differentiation protocol and has recently been published [38] showing the same expression pattern as hiPSC-CM EHTs. Atrial-like EHTs showed significantly higher expression for *MLC2A* and lower expression for *MLC2V* [26] (Fig. 6). Furthermore, atrial-like EHTs expressed higher levels of atrial specific genes like *SLN*, *ANP*, *COUP-TFI*, *COUPT-FII* and *PITX2* [24, 39–41]. The ventricular-specific *IRX4* gene [42] showed higher expression in ventricular-like EHTs than in atrial-like EHTs. Moreover, atrial selective ion channels encoding genes (*KCNA5*, *KCNJ3*, *SK2*, *SK3*) [43] were stronger expressed in the atrial-like EHTs compared to the ventricular-like EHTs (Fig. 6).

### Subtype-specific DNA methylation of hiPSC cardiomyocytes

**Fig. 7:**
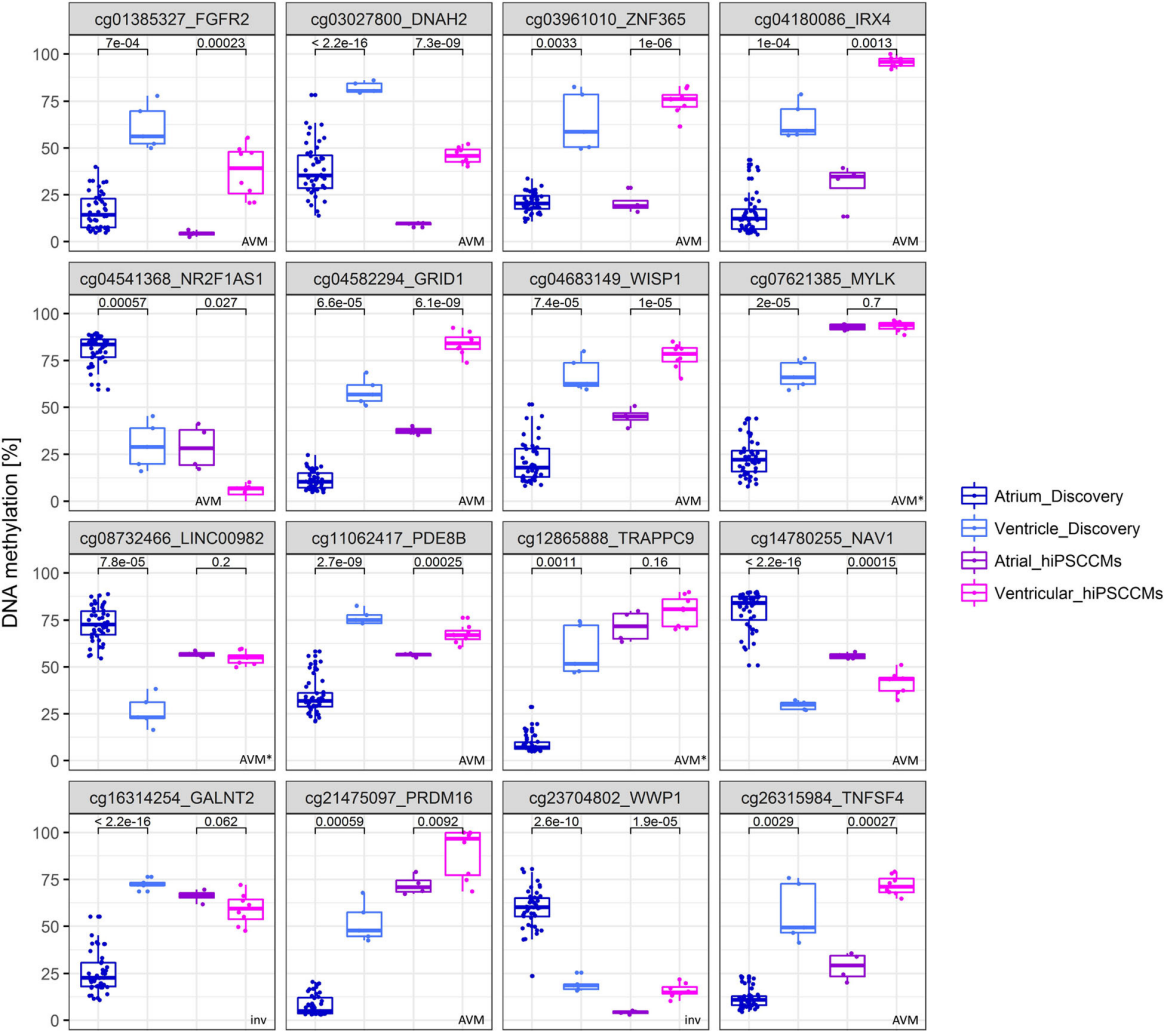
Bisulfite pyrosequencing analysis of candidate CpG loci in hiPSC-CMs: test of AVM pattern. The methylation values (percentage) of the initial discovery set (450K array analysis of atrial (*n* = 44, coloured dark blue) and ventricular (*n* = 5, coloured light blue) cardiac tissues from patients with CHDs) and in vitro-cultivated atrial and ventricular subtypes of hiPSC-CM 2D monolayers and 3D-engineered heart tissues (bisulfite pyrosequencing analysis, *n* = 4 atrial-like and *n* = 8 ventricular-like hiPSC-CMs; coloured dark violet and magenta, respectively) are depicted. CpG loci with significant (*p* < 0.05) differences in atrial and ventricular-like hiPSC-CMs showing similar AVM pattern as compared to the pattern in primary human atrial and ventricular cardiac tissues (discovery set) are marked with ‘AVM’. ‘Tendency loci’ with similar AVM pattern, that did not reach significance, are marked with ‘AVM*’ and loci showing opposite AVM pattern are marked with ‘inv’. Data is presented as standard box-and-whiskers plots (whiskers, 5th—95th percentile)

To investigate the heart tissue-predictive capability of the 16 candidate CpG loci, DNA methylation levels in different subtypes of hiPSC-CMs and -EHTs, batches of atrial and ventricular hiPSC-CMs from 2D monolayers (four ventricular-like (v)CM-2D and two atrial-like (a)CM-2D samples) and EHTs (four vCM-EHT and two aCM-EHT samples), were analysed using bisulfite pyrosequencing analysis. In total, 11 CpG loci showed similar AVM pattern with significant (up to *p* = 6.1 × 10^−9^) differences in atrial and ventricular-like hiPSC-CMs as compared to the pattern in primary human atrial and ventricular cardiac tissues (Fig. 7). These 11 CpG loci were associated with the following genes: *IRX4*, *GRID1*, *WISP1*, *TNFSF4*, *FGFR2*, *PRDM16*, *DNAH2*, *ZNF365*, *PDE8B*, *NR2F1-AS1* and *NAV1*. DNA methylation differences ranged from Δ%-methylation = 11.1 to Δ%-methylation = 65.2 with 64% of loci having a Δ%-methylation ≥ 32. Three CpG loci (*TRAPPC9* (cg12865888), *MYLK* (cg07621385) and *LINC00982* (cg08732466))—hereinafter referred to as ‘tendency-loci’—showed similar AVM patterns in hiPSC-CMs when compared to cardiac tissue samples but did not reach statistical significance (Fig. 7). Two CpG loci—at *WWP1* (cg23704802) and *GALNT2* (cg16314254, albeit not significant)—showed inverse AVM patterns in hiPSC-CM 2D monolayers and 3D EHTs as compared to human cardiac tissue samples. No differences were detected between atrial or ventricular hiPSC-CMs from 2D versus 3D format. All in all, among the 16 tested CpG loci, we could show a concordance of 69% of heart tissue type classification in atrial and ventricular-like hiPSC-CMs, with 88% concordance when including the ‘tendency-loci’. Furthermore, DNA methylation at the 16 CpG loci was tested in three different cell lines—hiPSC-ECs (endothelial cells of the same hiPSCs as used for cardiac differentiation), valve interstitial cardiac cell line (VIC, Innoprot P10462) and MCF7 breast cancer cell line—to show specificity of AVM patterns for atrial and ventricular cardiac cells (Additional file 13: Figure S13). Overall, hiPSC-ECs mostly showed lower DNA methylation values than atrial or ventricular hiPSC-CMs. No clear DNA methylation pattern could be detected: while seven CpG loci revealed similar DNA methylation values ± 7% comparable to atrial hiPSC-CMs, the remaining CpG loci showed an intermediate DNA methylation status or either considerably lower or higher DNA methylation values as compared to atrial/ventricular hiPSC-CMs (Additional file 18: Table S5). Human cardiac VICs revealed very low DNA methylation values (median 9.4%) over all 16 CpG loci. MCF7 cell line showed high DNA methylation values (median 77.6%). All three cell lines—hiPSC-EC, VIC and MCF—did not resemble the AVM pattern over the 16 candidate CpG loci, neither in absolute DNA methylation values nor in a clear pattern by showing a tendency towards either atrial or ventricular heart tissue DNA methylation.

## Discussion

For appropriate cellular cardiac disease modelling, a decent phenotypic characterization of hiPSC-CMs is essential. Current methods to discriminate different cardiac cell types are mostly time-consuming and often provide imprecise phenotypic evaluation [21, 44]. Here, we identified highly significant differential DNA methylation patterns in atrial compared to ventricular human cardiac tissue samples by array-based genome-wide DNA methylation analysis. Systematic analysis of a small set of defined CpG loci enabled us to reproducibly detect atrial and ventricular tissues of independent sampled cohorts and to distinguish atrial and ventricular subtypes of hiPSC-CMs.

By analysing genome-wide DNA methylation profiles in different anatomical regions of the human heart using a discovery set of 49 cardiac tissue samples, we identified distinct differential DNA methylation patterns in atrial and ventricular primary cardiac tissues using the 450K array platform. Up to now, only a few studies exist, analysing the DNA methylation in human heart tissue [12–14, 45]. Especially, the limited availability of primary heart tissues and the small amounts of material excised routinely during surgery are challenging aspects in such studies. The presented data of this study are—to our knowledge—the first revealing distinct DNA methylation patterns in different human cardiac tissues. We were able to detect exceedingly high delta *β*-values (mean Δ*β* = 0.4) among the 168 significantly differentially methylated CpG loci (*q* ≤ 1 × 10^−6^, σ/σ_max_ > 0.4) in atrial compared to ventricular cardiac tissues. The highly differential atrial-ventricular methylation (AVM) pattern of the 168 CpG loci might reflect an important influencing factor for cardiac tissue differentiation. In order to investigate if the atrial and ventricular cardiac tissues showed any differences in the expression of DNA methyltransferases, qPCR analyses of *DNMT1* and *DNMT3A* were performed. While the expression of *DNMT1* showed no significant differences, *DNMT3A* revealed significantly higher expression in atrial tissue compared to ventricular tissue (Additional file 7: Figure S7). This might be due to individual effects of the analysed sample, as previous GTEx [46] experiments showed no significant differences in expression of *DNMT3A* between atrial and ventricular tissue, and studies in mice revealed that deficiency of de novo CpG methylation capacity mediated by DNMT3A and DNMT3B was dispensable for pathological mechanisms in heart failure [47]. Unfortunately, no additional tissue material was available from cardiac samples analysed in this study to perform further analyses of changes in the activity of the DNA methyltransferases.

Although the 168 differentially methylated CpGs were not associated to TFBSs of any specific cardiac TFs, developmental (cell specific) TFs like GATA2, FOS and TCF7L2 were among the ten most common TFs (Additional file 6: Figure S6). Also, over-representation analysis by Reactome Pathways Tool [33] resulted in significant enrichment in the pathway ‘Developmental Biology’ (R-HSA-1266738, *q* = 6.3 × 10^−3^) of genes with differentially methylated CpGs and associations to TFBSs. This indicates that the differential DNA methylation of the 168 CpGs is associated with—and might influence—developmental processes involved in differentiation towards atrial and ventricular cells. DNA methylation is not necessarily associated with the inhibition of TF binding [48], and to further elucidate the impact of methyl-TFBSs on atrial and ventricular differentiation processes, prospective investigations have to be done in a cell specific manner in vivo.

The 168 CpG loci did not overlap with known age-related CpGs [49] or DMRs (differentially methylated regions) identified in epigenome-wide association scans of age or with age-related phenotypes according to Bell et al. (2012) [50], so that, with reasonable certainty, we could exclude the possibility of age-related methylation effects. However, until now, only limited data is available regarding age-related changes in human heart tissues, and in the study of Horvath [49], the heart tissues showed a relatively low correlation of predicted DNA methylation age and chronological age. Therefore, we performed an in-depth literature research which thus far did not reveal any of the 168 CpG loci to be associated with gender or age effects.

Considering regulatory features, a significant enrichment (OR = 3.6, *p* = 5.9 × 10^−16^) of CpGs overlapping with predicted enhancer elements determined by ENCODE Project Consortium [31] (50% of CpGs) could be shown (Additional file 5: Figure S5). As enhancers are key regulatory elements that control the process of establishing a tissue-specific transcriptional programme and can be regulated by DNA methylation [51], this might indicate a relation of differential DNA methylation in differentiation processes towards atrial or ventricular tissues. Although only a few cardiac enhancers have thus far been identified, being less evolutionarily defined than other tissue-specific enhancers [52], we could identify 24 CpG loci overlapping with predicted human heart enhancers from a study of Dickel et al. [35] which underpins the hypothesis of differential DNA methylation-driven atrial and ventricular tissue differentiation. However, enrichment of regulatory features and certain gene pathways in 450K array data has to be regarded with caution. Selection of loci of 450K array was defined by a set of content categories identified by a consortium of epigenetics researchers [30], a design that is biassed due to preselection of probes that interrogate only certain CpG sites, therefore, the design is not hypothesis neutral. For future comprehensive studies of all heart tissue-specific differentially methylated loci, whole genome bisulfite sequencing would be the ideal method as it best represents regions of lower CpG density (e.g. intergenic ‘gene deserts’ or distal regulatory elements) that potentially control tissue-specific gene expression [53].

Methylation data of dynamic changes in the cardiac methylome during postnatal development are available and in order to compare changes in DNA methylation potentially involved in developmental and differentiation processes, the MBD-Seq data of Sim et al. on cardiac left ventricle of mice [34] was compared to the 450K data of the present study. Previous analyses of Zhou et al. (2017) of cross-species DNA methylation (rat, mouse, human) revealed that a significant proportion of tissue-specific DNA methylation is conserved [54] and considering that protein-coding genes and gene-regulatory regions (both genomic regions with CpG loci primarily targeted by 450K array) show high sequence similarities between mouse and human [55] [56] [57], informative results were expected. However, even with less stringent approaches, only one CpG locus, cg04115185, laying in a highly conserved non-coding region, showed overlap to one MBD-Seq read (left ventricle of mouse P1). This CpG locus was hypermethylated in ventricular tissue compared to atrial tissue in the present study. If one regards the technique of MBD-Seq, capturing hypermethylated regions, cg04115185 exhibited the same DNA methylation in mouse and human left ventricle in a conserved non-coding region with *IRX4* as its nearest gene (390 kb distance). A high fraction of sequences that are conserved across multiple species resides in non-coding regions [58] and might be associated with the control of early development [59] and tissue-specific gene expression [53]. Therefore, differential DNA methylation of cg04115185 might potentially be involved in regulation of the ventricular-specific *IRX4* gene [42] which plays an important role in regulating chamber-specific gene expression in the developing heart [60, 61]. All in all, the little overlapping of the two data sets could either be due to differences in DNA methylation analysis platforms, as MBD-Seq is a Methyl-CpG-binding domain-based capture method biassed towards hypermethylated regions [62], or due to cross-species differences between mouse and human concerning DNA methylation patterns. However, no study exists yet, to our knowledge, that investigates DNA methylation in primary human heart tissue from neonates and infants as it was conducted in the present study.

Further bisulfite pyrosequencing analyses enabled us to identify 16 CpG loci allowing for reproducible verification of the AVM pattern. Given that heart tissue samples are rather difficult to obtain and only small amounts of material are excised routinely during surgery, we could only use the remaining sample material of seven atrial and four ventricular samples (verification set) from the discovery set to verify the 450K array results using bisulfite pyrosequencing. The 16 candidate CpG loci showed heart tissue-predictive capability as we could consistently detect the AVM pattern in independently sampled cardiac tissues. These cardiac tissues consisted of pathologic tissues from cardiac patients (CHDs, HF) and this may affect the results, which is why we also included cardiac tissues from non-transplantable heart-healthy donor hearts (validation set). Although we could partially detect variations in absolute methylation values between sample cohorts (discovery, validation and verification set) that might be due to differences in clinical phenotypes or technical issues, all 16 candidate CpG loci exhibited significant DNA methylation differences between atrial and ventricular tissues in each sample cohort. This tissue-predictive capability of the 16 CpG loci was even valid in the non-pathologic samples from non-transplantable heart-healthy donor hearts and therefore occurring irrespectively from cardiac phenotype. Due to the small amounts of material excised routinely during surgery no further cell sorting was possible, therefore our analyses are based on bulk cardiac tissue samples. These comprised mostly the muscular parts, the myocardium. The human heart contains many different cell types, while the volume fraction of the heart occupied by CMs accounts for 70–80% [63]. We therefore assumed that our methylation patterns represent cardiac tissue type-specific signatures reflecting the morphological and functional differences of atrial and ventricular myocardium and predominantly their CMs. In particular, with respect to electrophysiological and contractile properties, atrial and ventricular CMs differ significantly [64]. Considering the many different epigenetic processes that have been implicated in influencing cardiac gene expression in development and disease [11, 14, 65, 66], the observed differential AVM pattern might represent an epigenetic signature for differentiation of atrial and ventricular subtypes of CMs.

We hypothesized that the AVM pattern in cardiac tissues could also be identified in atrial and ventricular-like subtypes of in vitro-derived hiPSC-CM populations, allowing us to distinguish hiPSC-CMs by their DNA methylation. Differentiated in the absence of retinoic acid, hiPSC-CMs from 2D monolayers and 3D EHTs showed a phenotype resembling (immature) ventricular CMs, since we detected the ventricular-specific marker MLC2v [26]. Further evidence for their ventricular phenotype is provided by electrophysiological lack of increase in *I*_m_ by the acetylcholine analogue carbachol [28] as acetylcholine-activated potassium currents typically exist in atrial and not in ventricular tissue [67]. Besides ventricular-like hiPSC-CMs, we were able to differentiate atrial-like hiPSC-CMs by activating retinoid signals [68] during cardiac specification based on published protocols [24, 25, 39]. The impact of retinoic acid on cardiomyocyte subtype specification in hiPSC-CM differentiation is demonstrated by differential expression of a set of established marker genes (Fig. 6). The higher expression of atrial natriuretic peptide, *SLN*, *MLC2A*, *COUP-TFI*, *COUP-TFII*, *KCNA5*, *KCNJ3*, *SK2* and *SK3* genes indicates an atrial phenotype [24, 39–41, 43]. In addition to expression analyses, we investigated the potential of using 16 candidate CpG loci to detect the cellular identity of atrial and ventricular-like cells of in vitro-derived hiPSC-CMs by testing their methylation profiles. We observed significant differential AVM patterns at 11 CpG loci in atrial and ventricular-like hiPSC-CM subtypes, reflecting similar methylation signatures of human atrial and ventricular tissue. In order to verify specificity of AVM patterns of the 16 candidate CpG loci, we tested the DNA methylation using bisulfite pyrosequencing in further cell lines which included endothelial cells from the original hiPSC line (hiPSC-EC), human cardiac VICs and a non-cardiac cell line MCF7 (breast cancer cell line). In hiPSC-ECs, seven CpG loci revealed similar DNA methylation values ± 7% comparable to atrial hiPSC-CMs (Additional file 13: Figure S13) which could be explained by their same hiPSC origin. All in all, there was no concordance between DNA methylation values of hiPSC-ECs and atrial/ventricular heart tissue. The remaining CpG loci though showed considerably lower or higher DNA methylation values or intermediate DNA methylation status in hiPSC-ECs as compared to aCMs/vCMs and atrial/ventricular tissues, respectively. Hence, no DNA methylation pattern could be detected in hiPSC-ECs that would recapitulate the AVM pattern. The same applied to MCF7 cell line which showed very high DNA methylation values (median 77.6% over the 16 CpG loci) or human cardiac VICs with low DNA methylation values (median 9.4%)—both revealing no correlation to atrial or ventricular heart tissue DNA methylation. All in all, the observed AVM patterns appear to be specific for atrial and ventricular heart tissues. It is noteworthy that additionally, three CpG loci (located on *TRAPPC9*, *MYLK* and *LINC00982*), although not significant, showed also comparable AVM patterns in hiPSC-CMs. The slight but not significant DNA methylation differences between atrial and ventricular-like hiPSC-CMs at these three loci and the inversely methylated CpG loci at *GALNT2* and *WWP1* (opposite AVM patterns of hiPSC-CMs in comparison with cardiac tissue samples) might be due to the characteristics of in vitro-generated hiPSC-CMs, which are not to be equated with native myocardium and do not show a fully mature phenotype [69]. Cardiac maturation has long been investigated in numerous studies [70–72]. Nevertheless, the identification of techniques to differentiate hiPSC-CMs to mature cardiomyocytes is only in its initial stages [69, 73]. Therefore, deviations from the observed AVM patterns could be explained by the differences in the degree of differentiation between hiPSC-CMs and native myocardial samples. However, on account of the largely highly significant DNA methylation differences measured in atrial and ventricular-like hiPSC-CM subtypes showing similar AVM patterns as in human cardiac tissues, these 11 CpG loci could represent potential loci for subtype characterization of in vitro-generated cardiomyocytes. Taking into consideration that previous techniques for phenotyping of hiPSC-CMs are mostly time-consuming approaches, testing DNA methylation of only a small set of CpG loci might provide a rapid and cost-effective option to identify the differentiation state of in vitro-generated cardiomyocytes. Compared to this, immunofluorescence stainings (fixation, antibody incubations) [26, 74] and electrophysiological techniques (e.g. action potential (AP) and ionic currents) [23, 27, 28] of hiPSC-CMs are laborious and costly proceedings. Differentiation protocols and cell culture conditions may influence AP phenotypes of hiPSC-CMs allowing merely imprecise phenotypic evaluation [75] [76] using this electrophysiological technique. Analyses of the expression of key structural and functional genes in hiPSC-CMs using qPCR is a standard method of hiPSC-CM molecular profiling [23]. One could argue that the method of bisulfite pyrosequencing we put forward here is just as time-consuming as qPCR techniques since both require pretreatment of starting material. But unlike DNA which is used for bisulfite sequencing, RNA is severely delicate once extracted from its cellular environment and the linearity of the reverse-transcription step to create cDNA may be unsteady, as secondary structures and primer-independent cDNA synthesis can influence the outcome [77] [78]. In general, targeting the genome and its modifications like DNA methylation results in robust data [79] while transcriptome data is context-dependent with varying mRNA complement and level depending on physiological state and changes in cell culture conditions [77]. Therefore, determining the DNA methylation in few candidate CpG loci might provide a valuable alternative to molecularly profile in vitro-generated cardiomyocytes.

## Conclusions

In conclusion, we have investigated the genome-wide DNA methylation pattern of human cardiac tissues from different anatomical regions of the heart. We subsequently assessed the DNA methylation level at candidate CpG loci in further independently sampled cardiac tissues and in vitro-generated hiPSC-CM subtypes. We identified distinct differential DNA methylation patterns in atrial compared to ventricular human cardiac tissues. A key finding of our study is the potential of using a small number of candidate CpG loci allowing for lineage commitment verification of atrial and ventricular-like hiPSC-CM subtypes. We showed that the current hiPSC lines do not fully recapitulate the epigenetic DNA modification of human atrial and ventricular heart tissue. However, our method is applicable to guide this process, enabling to distinguish cardiac tissue subtypes by analysing only few CpG loci. Thus, this method might serve as a rapid approach for characterization of in vitro-generated cardiomyocytes, potentially improving prospective research of hiPSC-CMs and patient-specific therapy.

## Methods

### Human heart tissue samples

Heart tissue samples were obtained from paediatric patients with congenital heart disease (CHD) and from adult patients with arrhythmic heart defects as well as from non-failing (NF) heart samples of ejected donor hearts which could not be transplanted for technical reasons. Interatrial septum (IAS) samples were obtained from 25 patients with hypoplastic left heart syndrome (HLHS) and from nine patients with hypoplastic right heart syndrome (HRHS), including tricuspid valve atresia (TA). Furthermore, samples from the right atrium (RA) of one patient with HLHS and of three patients with atrioventricular septal defect (AVSD) were obtained during open-heart surgery. The tissue samples were routinely excised in the first weeks of life during surgery and immediately snap-frozen in liquid nitrogen, ensuring an ex vivo time of less than 5 min. Care was taken to use mainly the muscular part rather than the endocardial part of the cardiac samples in this study. Heart explants from one patient with HLHS and transposition of the great arteries (TGA) and from one patient with left ventricular aneurysm (LVA) were available to excise tissue samples (biological triplicates and duplicates) from the myocardium of left and right atrium (LA, RA) and left and right ventricle (LV, RV). Furthermore, IAS, LA and LV tissue samples could be obtained postmortem (12 hpm) from one patient with familial dilated cardiomyopathy (DCM). Myocardial samples from LA and RA from adult patients with heart failure (HF) were obtained to additionally analyse heart tissue from adult donors. Moreover, LA, RA, LV and RV samples from seven non-transplantable donor hearts were investigated to include non-failing heart samples. A table listing the type of heart tissue, diagnosis, gender and age of patients and controls is given in the supplementary data (Additional file 14: Table S1).

### Cultivation of hiPSC-derived cardiomyocytes and engineered heart tissues

Expansion of undifferentiated human-induced pluripotent stem cells (hiPSCs), cardiomyocyte (CM) differentiation and generation of 3D-engineered heart tissues (EHTs) were performed as recently described [80]. In brief, expansion of undifferentiated hiPSCs was performed in FTDA medium. Embryoid body (EB) formation was induced in stirred suspension cultures (spinner flasks). Mesodermal induction was achieved using BMP-4 (10 ng/ml), activing A (3 ng/ml) and bFGF (5 ng/ml) in the absence of insulin in RPMI medium [80]. Specification of cardiac differentiation of mesodermal progenitors was performed by WNT signal inhibition (XAV939, 1 μM). This resulted in a population of a primarily ventricular cardiomyocyte (ventricular-like) phenotype. Based on previous reports [25, 39] differentiation of atrial cardiomyocytes was achieved by addition of retinoic acid (1 μM) for the first 3 days of Wnt signalling inhibition. By fluorescence-activated cell sorting (FACS), hiPSC-CM differentiation efficiency of cardiac troponin T positive cells was analysed to ensure that similar differentiation efficiency could be obtained in the absence and presence of retinoic acid. At the end of cardiac differentiation, EBs were enzymatically dispersed with collagenase [80]. The dissociated cells were mixed with fibrinogen (Sigma F4753) and thrombin (100 U/ml, Sigma Aldrich T7513) to generate EHTs (1 × 10^6^ cells/EHT) [80], a synchronously beating syncytium of hiPSC-CMs in two elastic silicone posts [26, 80].

### Phenotypic characterization of atrial and ventricular hiPSC cardiomyocytes

In order to characterize cultivated atrial and ventricular-like subtypes of hiPSC-CMs (2D cultures and EHTs), expression analyses of atrial and ventricular-specific genes were performed using quantitative real-time PCR (qPCR) [80] technique. Subtypes of hiPSC-CMs were maintained under standard cell culture conditions for 2 weeks before performing qPCR. After proteinase K (Thermo Scientific) digestion, extraction of total RNA was performed with RNeasy Mini Kit (Qiagen) according to the manufacturer’s instructions. qPCR experiments were performed on atrial and ventricular hiPSC-CMs from three independent hiPSC-CM generations, each as duplicates. For assessing gene expression by qPCR, cDNA was synthesized from approximately 200 ng of total RNA. RNA was reverse-transcribed into cDNA using high-capacity cDNA reverse transcription kit (Applied Biosystems). qPCR was performed using Maxima SYBR Green/ROX (Thermo Scientific) on an ABI Prism instrument (Applied Biosystems). Each reaction was performed in triplicates and non-template reaction (replacing cDNA with water) was used as negative control. The cycling parameters were 50 °C for 2 min followed by 95 °C for 10 min, 15 s at 95 °C and 1 min at 60 °C for 40 cycles. mRNA-specific CT values were normalized with CT values for human *GUSB* (beta glucuronidase). Relative differences between atrial and ventricular samples were calculated with ∆∆Ct method for relative quantifications. Primer sequences are enclosed in the Additional file 17: Table S4. Candidate markers for atrial and ventricular phenotype were chosen based on previous publications [81]. Statistical analyses were performed with GraphPad Prism software 5.0. Data are expressed as mean ± SEM in bar graphs. Differences between groups were analysed by unpaired *t* test. Results were considered statistically significant if the *p* value was less than 0.05.

### Isolation of DNA

Genomic DNA from frozen heart tissue samples and cardiomyocyte cell lines (2D monolayer, 3D EHT) was extracted using Gentra Puregene DNA isolation reagents (Qiagen) according to the manufacturer’s protocol (5–10 mg tissue). Following fluorometric quantification by Qubit dsDNA BR Assay (Life Technologies), DNA integrity was visually inspected by agarose gel electrophoresis.

### Illumina Infinium HumanMethylation450 Assay

The DNA methylation analysis of human heart tissue samples was conducted using the Infinium HumanMethylation450 BeadChip (Illumina), which interrogates the methylation level of 485,577 loci. Bisulfite conversion of isolated DNA (1 μg) was performed using the Zymo EZ DNA Methylation Kit (Zymo Research) according to the manufacturer’s instructions. Bisulfite-converted DNA was eluted in 15 μl ddH_2_O. Isothermal amplification, enzymatic fragmentation, hybridization (for 20 h) onto the HumanMethylation450 Bead Chips (Illumina) and subsequent scanning of immunohistochemistry staining using an iScan Microarray Scanner (Illumina) were performed following the manufacturer’s protocol as described earlier [30]. Samples were randomly distributed across arrays to limit batch effects.

### Processing and quality control of Illumina Infinium HumanMethylation450 data

Signal intensities and raw methylation values were extracted from the GenomeStudio™ Software (version 2011.1, Methylation Analysis Module version 1.9.0, Illumina) for each CpG without any data processing. Methylation levels (*β*-values) are given as ratios of fluorescent signal intensities between methylated and unmethylated alleles, ranging from *β* = 0 (unmethylated) to *β* = 1 (completely methylated). Hybridization quality was analysed using detection *p* values calculated by the GenomeStudio™ Software for each CpG. To estimate the percentage of loci showing a median detection *p* value < 0.01 for each sample, a loci call rate (LCR) was calculated as LCR = ((number of loci with detection *p* value < 0.01)100) (485,577 total number of loci)^−1^. Samples with LCR > 98% were included in subsequent data processing. Next, 450K array data was normalized using the normalization function of RnBeads [82] R package, a *Subset*-*quantile Within Array Normalization* (SWAN) method. Following this, the data was filtered by (1) probes mapping to sex chromosomes, to avoid gender specific bias; (2) probes harbouring SNPs with an allele frequency (AF) > 0.05 as reported by 1000G rel. 20110521; (3) probes comprising annotated SNPs (1000G rel. 20110521) within 3 bp of the interrogated CpG having an AF > 0.05; and (4) cross-reactive probes according to Chen at al. [83]. Following these preprocessing steps, *β*-values of biological replicates from one patient with HLHS-TGA (3× LV, 3× RV, 3× LA, 3× RA) and one patient with LVA (3× LV, 2× RV, 3× LA, 3× RA) were combined to average values each. Thus, the final 450K dataset consisted of 44 atrial heart datasets (35× IAS, 3× LA, 6× RA) and five ventricular datasets (3× LV, 2× RV).

### Statistical analysis

Unsupervised principal component analyses (PCA) of preprocessed and quality filtered 450K methylation data was performed using R statistical software [84] to obtain an overview of preliminary data. To identify differences in DNA methylation of distinct cardiac tissue types (IAS, RA, LA, RV, LV), analysis of variance (ANOVA) test with Benjamini-Hochberg [85] FDR multiple testing correction was applied using OMICS explorer (version 2.3, Qlucore) software. A variance filter of σ/σ_max_ > 0.4 was applied. To keep the number of false positives as small as possible, a stringent FDR of < 1 × 10^−6^ was used. The ANOVA was plotted as PCA using OMICS explorer (version 2.3, Qlucore) for 3D plots to visualize the segregation pattern of different cardiac tissue types and to analyse their epigenetic distance and relatedness. Based on these preliminary results, due to the observed distribution pattern, a Student’s *t* test was applied to compare the two segregating groups—atrial versus ventricular heart tissue samples using OMICS explorer (version 2.3, Qlucore) software. Showing a multiple test adjusted FDR of *q* < 1 × 10^−6^ and *σ*/*σ*_max_ > 0.4, CpG loci were considered being significantly differentially methylated between atrial and ventricular samples. Following this pairwise group comparison analysis, a hierarchical cluster analysis (heat map depiction) was applied to visualize the statistical results using OMICS explorer (version 2.3, Qlucore) software. To minimize effects due to intra-group heterogeneity and small group sizes, Welch two-sample *t* test was applied to analyse the significance of differential methylation of the 16 candidate CpG loci in the different analysis cohorts (Additional file 19: Table S6). Group data was compared using unpaired Welch two-sample *t* tests and were presented as standard box-and-whiskers plots (whiskers, 5th–95th percentile). A *p* value of *p* < 0.05 was considered to be statistically significant. Graph Pad Prism 5 (GraphPad Software, San Diego, CA, USA) was used for data analysis of qPCR experiments.

MBD-Seq data of DNA methylation in heart tissues during postnatal development of mice [34] was compared to the 450K data of the present study, in order to investigate possible similarities between changes in DNA methylation potentially involved in developmental and differentiation processes. Over 60 million reads of MBD-Seq data from P1 (GSM1462877) and P14 (GSM1462880) mouse cardiac left ventricle were compared to the 450K array target sequences (± 60 bp flanking the CpG) of 168 differentially methylated CpGs in atrial and ventricular human heart tissues. In a second step, a less stringent approach was conducted by comparing ± 10 bp around CpG sites of MBD-Seq query sequences to the 450K target sequences.

Furthermore, the correlation between methylation values measured by 450K array (discovery set) and those measured by bisulfite pyrosequencing (verification set) was analysed using linear regression statistics (method Pearson). For quality control of 450K array by means of reproducibility of measured *β*-values, the correlation between biological replicates was assessed and depicted in scatter plots using R statistical software [84]. Gene ontology analyses were performed using Reactome Pathways Tool [33] and PANTHER over-representation test [36] (PANTHER 14.0). Enrichment analyses were performed as follows: Fisher’s exact (*p* < 0.05) and multiple test adjustment by Benjamini & Hochberg method [85].

### Filtering for candidate CpG loci

In order to analyse further heart tissue samples regarding the aspect of differential DNA methylation between heart tissue from atrial and ventricular origin, candidate CpG loci were filtered to be subsequently analysed by bisulfite pyrosequencing technique. Methylation differences between atrial and ventricular tissues are presented as delta *β*-values (or Δ% methylation in case of bisulfite pyrosequencing data), as absolute values of atrial subtracted by ventricular methylation values.

To select candidate CpG loci, the 450K array data was filtered by (1) upper 10% quantile of CpG loci with greatest delta *β*-values (Δ*β*) among the 168 differentially methylated CpG loci between atrial (LA, RA, IAS) and ventricular (LV, RV) samples, identified by Student’s *t* test; (2) association of CpG to UCSC RefSeq gene region between TSS200 (0–200 bases upstream of the transcriptional start site) up to the 3′UTR; (3) if more than one CpG site among the upper 10% quantile is associated to the same UCSC RefSeq gene, then the one displaying the higher Δ*β*-value was chosen and the next significant CpG locus with high Δ*β*-value was included in the candidate CpG list; and (4) if no primer design using PyroMark Assay Design 2.0 software (Qiagen) was possible (e.g. due to high CpG density), the next significant CpG locus was selected according to the filtering method of (1) and (2), respectively.

### Bisulfite pyrosequencing of candidate CpG loci

To verify the 450K array data and to validate the methylation pattern in further heart tissue samples, bisulfite pyrosequencing was performed using a Pyromark Q96 ID sequencer (Qiagen). PyroMark Assay Design software (version 2.0, Qiagen) was applied for primer design (primer list Additional file 16: Table S3). To ensure methylation-independent amplification, primers were designed to hybridize with CpG-free sequences. Human high methylated genomic DNA (80-8061-HGHM5, EpigenDx) served as methylated control, and whole-genome-amplified DNA (WGA-DNA) served as unmethylated control. The WGA-DNA was prepared using a pool of ten female and male healthy DNA control samples, which was amplified using Illustra GenomiPhi™ V2 DNA Amplification Kit (GE Healthcare) and cleaned up using Wizard® DNA Clean-Up System (Promega). DNA samples were bisulfite converted using the Zymo EZ DNA Methylation Kit (Zymo Research) as aforementioned. Bisulfite-converted DNA (1 μl) was applied for PCR using PyroMark PCR Kit reagents (Qiagen). Biotinylated PCR products underwent washing and creation of single-strand structure by usage of the Vacuum Prep Tool (Biotage) and PyroMark Gold 96 Reagents Kit (Qiagen) according to the manufacturer’s instructions. Bisulfite pyrosequencing reactions and quantification of methylation (ranging from 0% to 100%) were performed on a Pyromark Q96 ID sequencer (Qiagen). Quality control included analysis of accordance of histogram and measured DNA methylation peak signals as well as inspection of DNA methylation values of methylated (> 70%) and unmethylated (< 10%) controls at analysed CpG loci.

## Additional files

Additional file 1: **Figure S1.** Principal component analysis of cardiac disease type among 448,814 loci. The segregation of *β*-values of 448,814 loci from 49 different cardiac tissue samples (mean values of biological replicates, for detailed information see Additional file 14: Table S1) that were subjected to 450K array DNA methylation analysis is depicted. Cardiac disease types are given in different colours and shapes: AVSD (red round dots), DCM (yellow triangles), HLHS (green solid squares), HRHS (blue crosses), LV-Aneurysm (purple squares). No differences in DNA methylation could be observed between different types of cardiac disease. (PDF 1442 kb)

Additional file 2: **Figure S2.** Principal component analysis of 450K array sentrix ID among 448,814 loci. The segregation of *β*-values of 448,814 loci from 49 different cardiac tissue samples (mean values of biological replicates, for detailed information see Additional file 14: Table S1) that were subjected to 450K array DNA methylation analysis is depicted. Sentrix IDs of different array slides are given in different colours and shapes as described in the figure legend. No batch effects due to sample distribution on the array occurred. (PDF 2126 kb)

Additional file 3: **Figure S3.** *β*-value correlation of cardiac tissue biological replicates. Linear regression analysis of *β*-values (448,814 loci per sample) from biological replicates (LA, RA, LV and RV; each triplicates or duplicates as shown in table) from CHD patients 0117 and 0126. All replicates showed high correlation of *β*-values (*R*^2^ > 0.99, *p* < 2.2 × 10^−16^). Scatter plots of two replicate combinations, RA1_0117-RA2_0117 and LV1_0126-LV2_0126, are depicted. (PDF 5245 kb)

Additional file 4: **Figure S4.** Unsupervised principal component analysis of *β*-values from 49 cardiac tissue samples. The segregation of *β*-values of 2045 CpG loci (*σ*/*σ*_max_ > 0.4) of 49 different cardiac tissue samples (IAS, LA, RA, LV, RV) that were subjected to 450K array analysis is depicted. Subgroups of atrial and ventricular samples are marked as dashed and solid edging, respectively. IAS: red spheres, LA: pink spheres, RA: green spheres, LV: blue spheres, RV: yellow spheres. Tendencies of segregation into two groups, atrial and ventricular cardiac tissues, could already be noticed in unsupervised analysis. (PDF 252 kb)

Additional file 5: **Figure S5.** Regulatory features and associations to UCSC gene regions among 168 differentially methylated atrial-ventricular CpGs. Regulatory features and UCSC gene regions of all 168 CpGs (‘overall’), of ventricular-hypo/atrial-hypermethylated CpGs (‘ventricle hypo/ atrium hyper’ *n* = 44 CpGs) and ventricular-hyper/atrial-hypomethylated CpGs (‘ventricle hyper/ atrium hypo’ *n* = 124 CpGs) of 450K array analysis are depicted. Highest percentage of overlap with regulatory features (ENCODE) could be shown in enhancer elements (ENH) (50% (84/168) of CpGs), CpG island shores (29% (48/168) of CpGs) and DNAse I hypersensitivity sites (DHS) with 14% (24/168). Associations to UCSC gene regions showed highest proportion in gene bodies (30% (51/168) of CpGs). Besides CpG island shores, which showed a higher proportion in ‘ventricle hypo/ atrium hyper’-CpGs as compared to ‘ventricle hyper/ atrium hypo’-CpGs, all other regulatory features or gene regions did not show any difference between heart tissues. Shore, 0–2 kb from of UCSC CpG island; shelf, 2–4 kb from UCSC CpG island. TSS200, 0–200 bases upstream of the transcriptional start site, TSS1500, 0.2–1.5 kb upstream of the TSS. DMR: differentially methylated region (RDMR: DMR in reprogrammed cells). Gene regions of the CpGs are given according to Illumina’s 450K array classifications and UCSC classifications. (PDF 299 kb)

Additional file 6: **Figure S6.** Transcription factor binding sites subject to differential DNA methylation among 168 CpGs. Analysis of 168 differentially methylated CpGs between atrial and ventricular heart tissue revealed an overlap of 54% (90/168) of CpG loci being associated to TFBSs in various cell lines from ChIP-seq experiments performed by ENCODE (Factorbook Motifs [32]). The ten most common TFs showing TFBSs among the 168 CpGs are labelled: POLR2A, EZH2, EP300, GATA2, FOS, RUNX3, CEBPB, TCF7L2, MAX and TEAD4. Only TFBSs with Cluster Scores (out of 1000) > 300 are listed (Additional file 15: Table S2). (PDF 354 kb)

Additional file 7: **Figure S7.** mRNA expression of DNA methyltransferase genes in atrial and ventricular tissues. qPCR experiments were performed on atrial (LA, RA; grey bars) and ventricular (LV, RV; white bars) tissues of one patient (0126) with LV-Aneurysm. Triplicates of cDNA samples were analysed. Expression data was normalized to *GUSB* housekeeping gene and compared to atrial or ventricular expression (∆∆ CT method), *p* < 0.05 (Student’s *t* test), bars show mean with SEM. (PDF 547 kb)

Additional file 8: **Figure S8.** Boxplots (*t* test) of 16 candidate CpG loci with differential atrial-ventricular methylation pattern. Differential DNA methylation (*β*-value) of 16 candidate CpG loci in atrial (*n* = 44) and ventricular (*n* = 5) cardiac tissue samples being either hypomethylated or hypermethylated in atrial tissue (coloured blue) compared to ventricular tissue (coloured red). Highly significant DNA methylation differences (*p* ≤ 0.0001) in atrial vs. ventricular tissues have been detected at 13 of 16 CpG loci (Welch Two Sample *t* test) with 9 loci displaying *p* values <7.76 × 10^−7^ (*p* values Additional file 19: Table S6). Data is presented as standard box-and-whiskers plots (whiskers, 5th–95th percentile). (PDF 3430 kb)

Additional file 9: **Figure S9.** Correlation between methylation values of 450K array and bisulfite pyrosequencing (*DNAH2*, *FGFR2*, *IRX4* and *LINC00982*). Linear regression analysis of methylation values (percentage) at four candidate CpG loci (on *DNAH2*, *FGFR2*, *IRX4* and *LINC00982*) from 11 cardiac tissue samples that have been subjected to 450K array analysis (*β*-values as percentage on *x*-axis) and bisulfite pyrosequencing (*y*-axis). The 11 samples of the verification set are listed in Additional file 14: Table S1. At all four candidate CpG loci a high correlation of the methylation values (*R*^2^ ranging from 0.98 to 0.99, and *p* values ranging from *p* = 1.7 × 10^−7^ to *p* = 8.2 × 10^−10^) from the two platforms, 450K array and bisulfite pyrosequencing, could be verified. (PDF 1201 kb)

Additional file 10: **Figure S10.** Correlation between methylation values of 450K array and bisulfite pyrosequencing (*GALNT2*, *GRID1*, *MYLK* and *NAV1*). Linear regression analysis of methylation values (percentage) at four candidate CpG loci (on *GALNT2*, *GRID1*, *MYLK* and *NAV1*) from 11 cardiac tissue samples that have been subjected to 450K array analysis (*β*-values as percentage on *x*-axis) and bisulfite pyrosequencing (*y*-axis). The 11 samples of the verification set are listed in Additional file 14: Table S1. At all four candidate CpG loci a high correlation of the methylation values (R^2^ ranging from 0.97 to 0.99, and *p* values ranging from *p* = 9.3 × 10^−7^ to *p* = 4.2 × 10^−9^) from the two platforms, 450K array and bisulfite pyrosequencing, could be verified. (PDF 1143 kb)

Additional file 11: **Figure S11.** Correlation between methylation values of 450K array and bisulfite pyrosequencing (*NR2F1-AS1*, *PDE8B*, *TRAPPC9* and *WISP1*). Linear regression analysis of methylation values (percentage) at four candidate CpG loci (on *NR2F1-AS1*, *PDE8B*, *TRAPPC9* and *WISP1*) from 11 cardiac tissue samples that have been subjected to 450K array analysis (*β*-values as percentage on x-axis) and bisulfite pyrosequencing (y-axis). The 11 samples of the verification set are listed in Additional file 14: Table S1. At all four candidate CpG loci a high correlation of the methylation values (*R*^2^ ranging from 0.97 to 0.99, and *p* values ranging from *p* = 9.3 × 10^−7^ to *p* = 6.9 × 10^−10^) from the two platforms, 450K array and bisulfite pyrosequencing, could be verified. (PDF 1184 kb)

Additional file 12: **Figure S12.** Correlation between methylation values of 450K array and bisulfite pyrosequencing (*PRDM16*, *TNFSF4*, *WWP1* and *ZNF365*). Linear regression analysis of methylation values (percentage) at four candidate CpG loci (on *PRDM16*, *TNFSF4*, *WWP1* and *ZNF365*) from 11 cardiac tissue samples that have been subjected to 450K array analysis (*β*-values as percentage on *x*-axis) and bisulfite pyrosequencing (*y*-axis). The 11 samples of the verification set are listed in Additional file 14: Table S1. At all four candidate CpG loci a high correlation of the methylation values (*R*^2^ ranging from 0.91 to 0.97, and *p* values ranging from *p* = 0.00026 to *p* = 3.7 × 10^−7^) from the two platforms, 450K array and bisulfite pyrosequencing, could be verified. (PDF 1164 kb)

Additional file 13: **Figure S13.** Bisulfite pyrosequencing analysis of candidate CpG loci: test of specificity of AVM pattern. DNA methylation patterns of different cell lines (grey bars, each single samples)—endothelial cells of the hiPSC line (hiPSC-EC), valve interstitial cardiac cell line (VIC, Innoprot P10462) and a non-cardiac cell line MCF7 (breast cancer cell line)—were investigated using bisulfite pyrosequencing technique to compare to the AVM patterns of cardiac tissues from patients with CHDs (discovery set, 450K array data of atrial (*n* = 44) and ventricular (*n* = 5) tissues; white bars) and *in vitro* cultivated atrial and ventricular subtypes of hiPSC-CMs (bisulfite pyrosequencing data, *n* = 4 atrial-like and *n* = 8 ventricular-like hiPSC-CMs; dotted bars). CpG loci with significant (*p* < 0.05) differences in atrial and ventricular-like hiPSC-CMs showing similar AVM pattern as compared to the pattern in primary human atrial and ventricular cardiac tissues (discovery set) are marked with ‘AVM’. ‘Tendency loci’ with similar AVM pattern, that did not reach significance are marked with ‘AVM*’ and loci showing opposite AVM pattern are marked with ‘inv’. hiPSC-ECs mostly showed lower DNA methylation values than atrial or ventricular hiPSC-CMs, human cardiac VICs revealed very low DNA methylation values (median 9.4%) over all 16 CpG loci and MCF7 cell line showed high DNA methylation values (median 77.6%). All three cell lines—hiPSC-EC, VIC, MCF—did not resemble the AVM pattern over the 16 candidate CpG loci. Data is presented as standard bar plots (mean with SEM). (PDF 6347 kb)

Additional file 14: **Table S1.** Phenotype data, sampling and analysis information. (XLSX 19 kb)

Additional file 15: **Table S2.** Methylation values of Infinium HumanMethylation450 BeadChip (450K) array analysis. (XLSX 235 kb)

Additional file 16: **Table S3.** Primers for bisulfite pyrosequencing. (XLSX 17 kb)

Additional file 17: **Table S4.** Primers for qPCR. (XLSX 13 kb)

Additional file 18: **Table S5.** Methylation values of bisulfite pyrosequencing analysis. (XLSX 22 kb)

Additional file 19: **Table S6.** *Welch Two Sample t* test comparing the methylation values from atrial and ventricular samples of the 16 candidate CpG loci. (XLSX 15 kb)

## Abbreviations

AVM: Atrial-ventricular DNA methylation
AVSD: Atrioventricular septal defect
CHD: Congenital heart defect
DCM: Dilated cardiomyopathy
EHT: Engineered heart tissues
HF: Heart failure
hiPSC-CM: Human-induced pluripotent stem cell-derived cardiomyocyte
HLHS: Hypoplastic left heart syndrome
HRHS: Hypoplastic right heart syndrome
IAS: Interatrial septum
LA: Left atrium
LV: Left ventricle
LVA: Left ventricular aneurysm
RA: Right atrium
RV: Right ventricle
TA: Tricuspid valve atresia
