# Supplementary figures and images for: DNA methylation profiling allows for characterization of atrial and ventricular cardiac tissues and hiPSC-CMs
Source: Clin Epigenetics. 2019 Jun 11;11:89. doi: 10.1186/s13148-019-0679-0 (PMC6560887; doi:10.1186/s13148-019-0679-0)

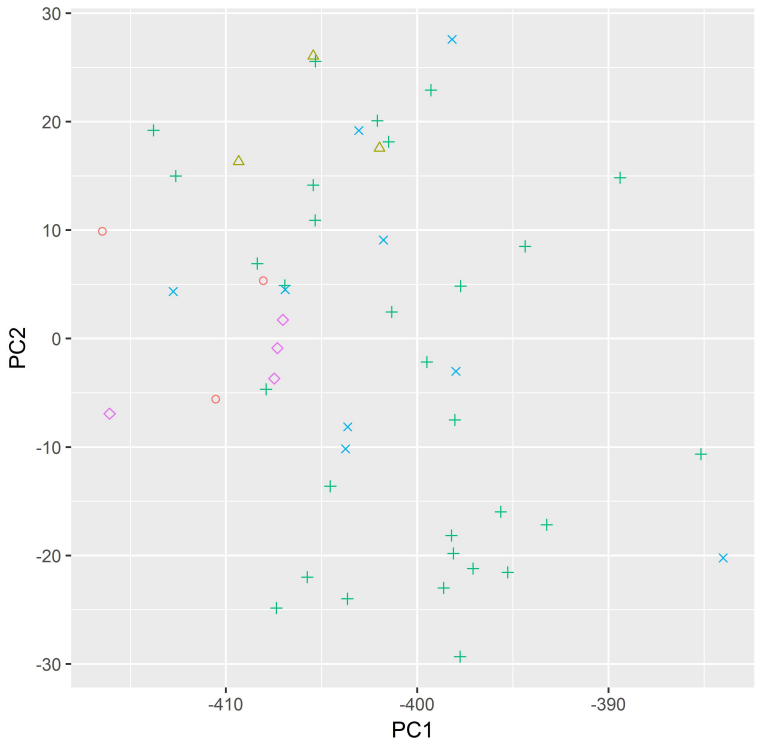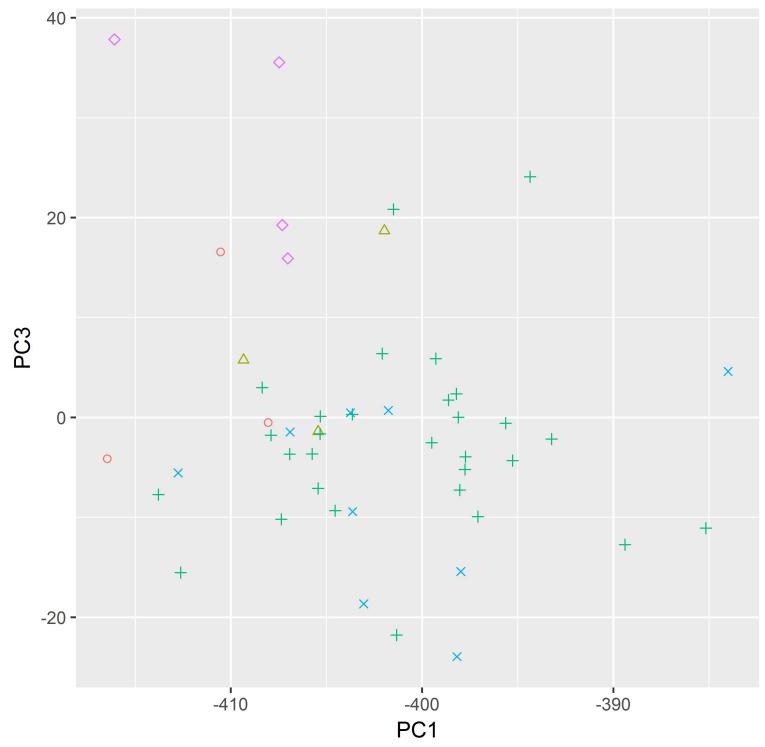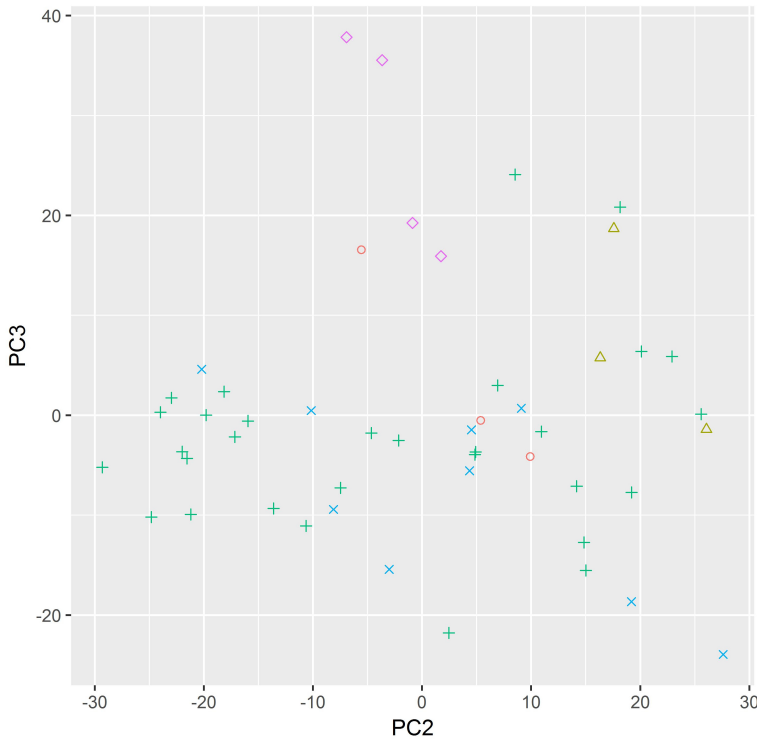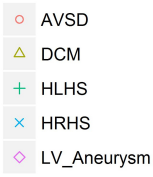

Supplement: Supplementary file 1 — Figure S1. Principal component analysis of cardiac disease type among 448,814 loci. The segregation of β-values of 448,814 loci from 49 different cardiac tissue samples (mean values of biological replicates, for detailed information see Additional file 14: Table S1) that were subjected to 450K array DNA methylation analysis is depicted. Cardiac disease types are given in different colours and shapes: AVSD (red round dots), DCM (yellow triangles), HLHS (green solid squares), HRHS (blue crosses), LV-Aneurysm (purple squares). No differences in DNA methylation could be observed between different types of cardiac disease. (PDF 1442 kb) [file 13148_2019_679_MOESM1_ESM.pdf]

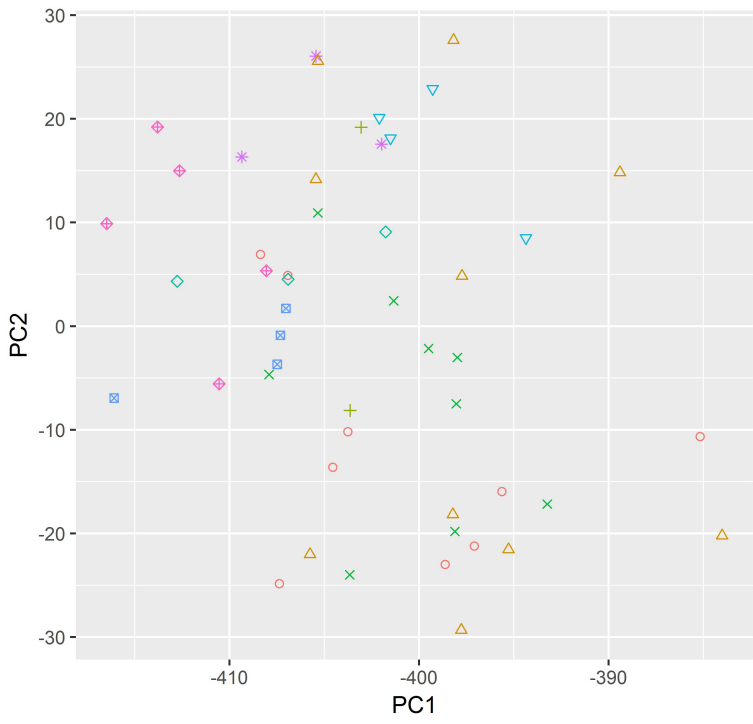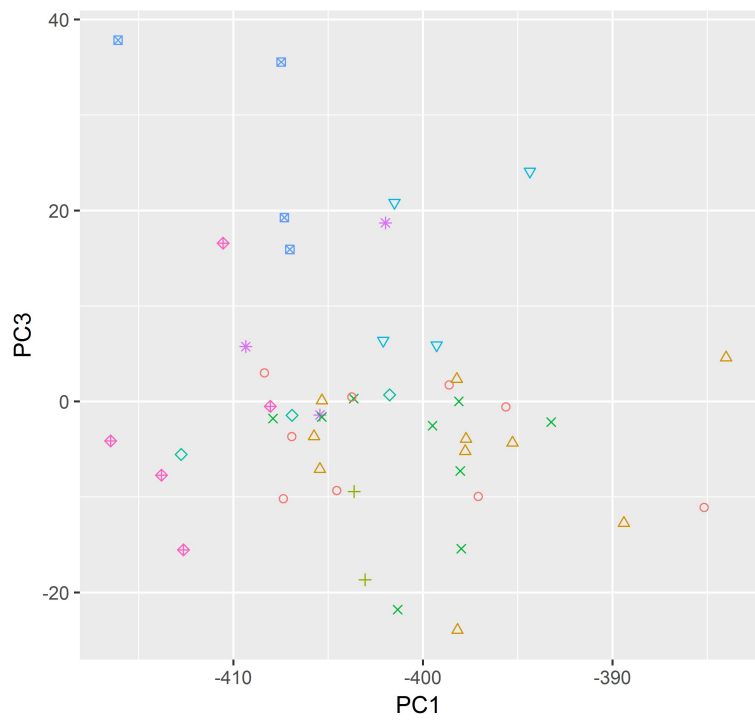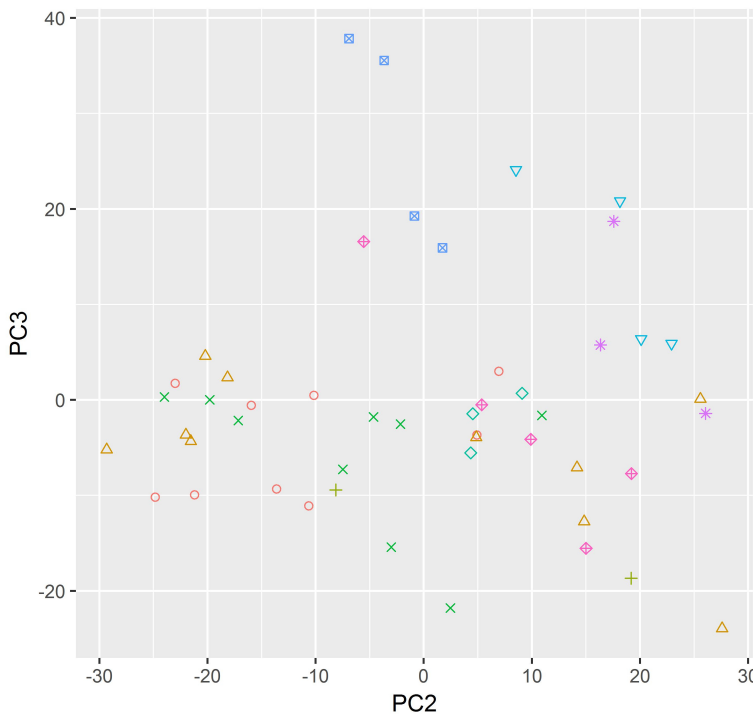

- sentrix\_7959804031
- △ sentrix\_7959804032
- + sentrix\_7959804046
- × sentrix\_7959804067
- ◇ sentrix\_7959804112
- ▽ sentrix\_8942342087
- ⊠ sentrix\_8942342111
- \* sentrix\_8942351095
- ⬠ sentrix\_8942351168

Supplement: Supplementary file 2 — Figure S2. Principal component analysis of 450K array sentrix ID among 448,814 loci. The segregation of β-values of 448,814 loci from 49 different cardiac tissue samples (mean values of biological replicates, for detailed information see Additional file 14: Table S1) that were subjected to 450K array DNA methylation analysis is depicted. Sentrix IDs of different array slides are given in different colours and shapes as described in the figure legend. No batch effects due to sample distribution on the array occurred. (PDF 2126 kb) [file 13148_2019_679_MOESM2_ESM.pdf]

○ Atrium  
○ Ventricle

■ IAS  
■ LA  
■ RA  
■ LV  
■ RV

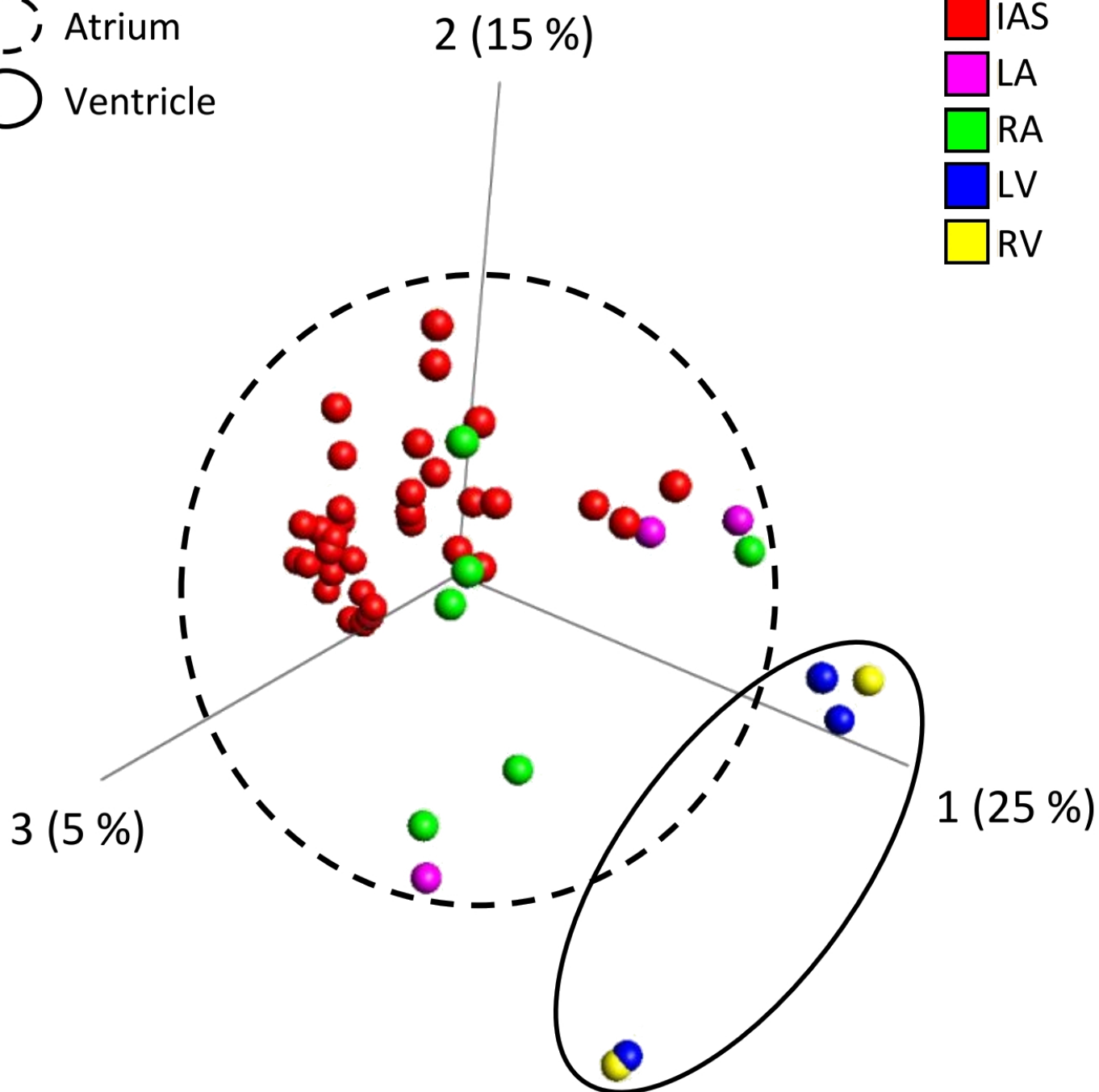

Supplement: Supplementary file 4 — Figure S4. Unsupervised principal component analysis of β-values from 49 cardiac tissue samples. The segregation of β-values of 2045 CpG loci (σ/σmax > 0.4) of 49 different cardiac tissue samples (IAS, LA, RA, LV, RV) that were subjected to 450K array analysis is depicted. Subgroups of atrial and ventricular samples are marked as dashed and solid edging, respectively. IAS: red spheres, LA: pink spheres, RA: green spheres, LV: blue spheres, RV: yellow spheres. Tendencies of segregation into two groups, atrial and ventricular cardiac tissues, could already be noticed in unsupervised analysis. (PDF 252 kb) [file 13148_2019_679_MOESM4_ESM.pdf]

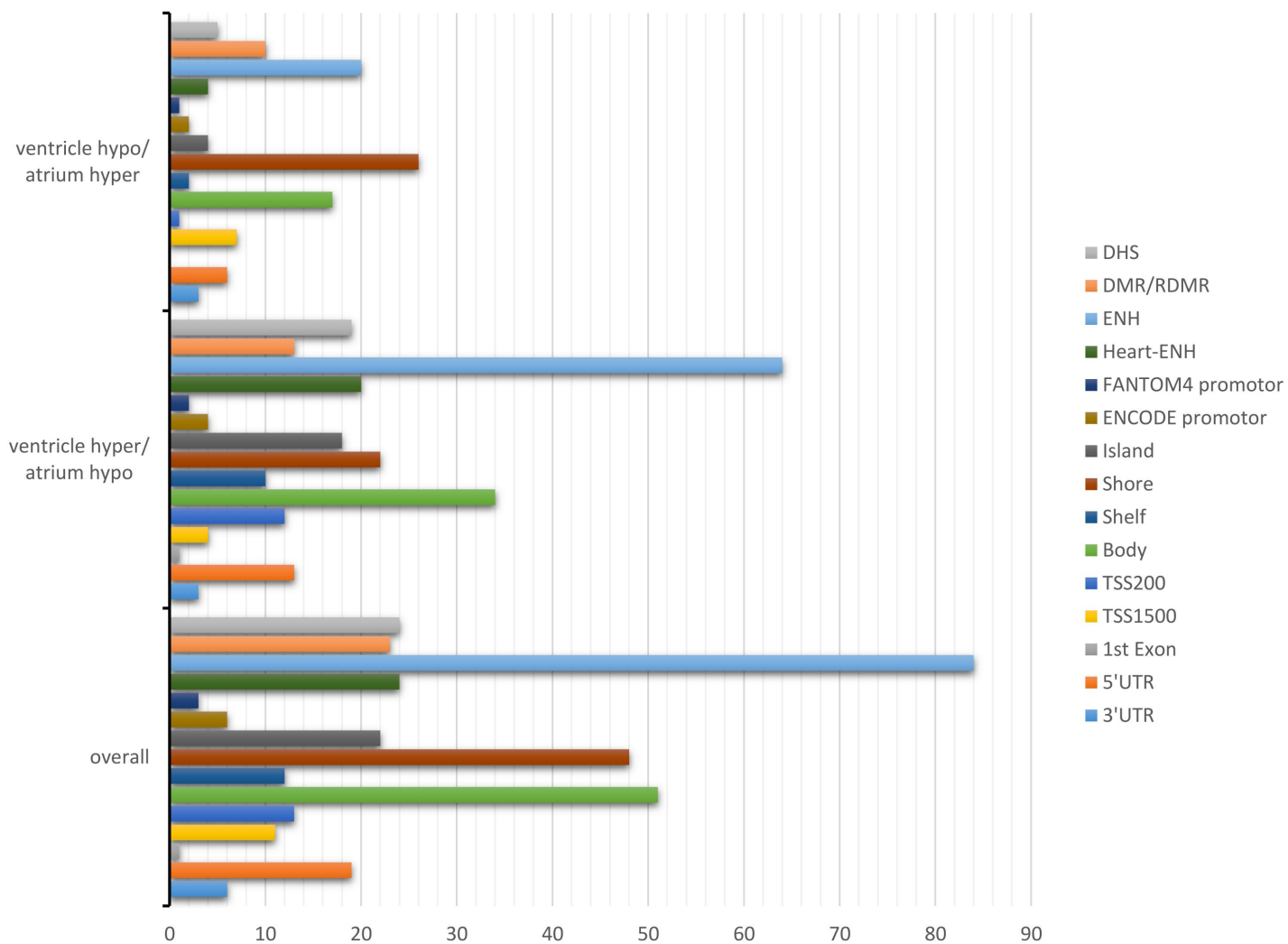

Number of associated CpGs within 168 diff. methylated atrial-ventricular CpGs

Supplement: Supplementary file 5 — Figure S5. Regulatory features and associations to UCSC gene regions among 168 differentially methylated atrial-ventricular CpGs. Regulatory features and UCSC gene regions of all 168 CpGs (‘overall’), of ventricular-hypo/atrial-hypermethylated CpGs (‘ventricle hypo/ atrium hyper’ n = 44 CpGs) and ventricular-hyper/atrial-hypomethylated CpGs (‘ventricle hyper/ atrium hypo’ n = 124 CpGs) of 450K array analysis are depicted. Highest percentage of overlap with regulatory features (ENCODE) could be shown in enhancer elements (ENH) (50% (84/168) of CpGs), CpG island shores (29% (48/168) of CpGs) and DNAse I hypersensitivity sites (DHS) with 14% (24/168). Associations to UCSC gene regions showed highest proportion in gene bodies (30% (51/168) of CpGs). Besides CpG island shores, which showed a higher proportion in ‘ventricle hypo/ atrium hyper’-CpGs as compared to ‘ventricle hyper/ atrium hypo’-CpGs, all other regulatory features or gene regions did not show any difference between heart tissues. Shore, 0–2 kb from of UCSC CpG island; shelf, 2–4 kb from UCSC CpG island. TSS200, 0–200 bases upstream of the transcriptional start site, TSS1500, 0.2–1.5 kb upstream of the TSS. DMR: differentially methylated region (RDMR: DMR in reprogrammed cells). Gene regions of the CpGs are given according to Illumina’s 450K array classifications and UCSC classifications. (PDF 299 kb) [file 13148_2019_679_MOESM5_ESM.pdf]

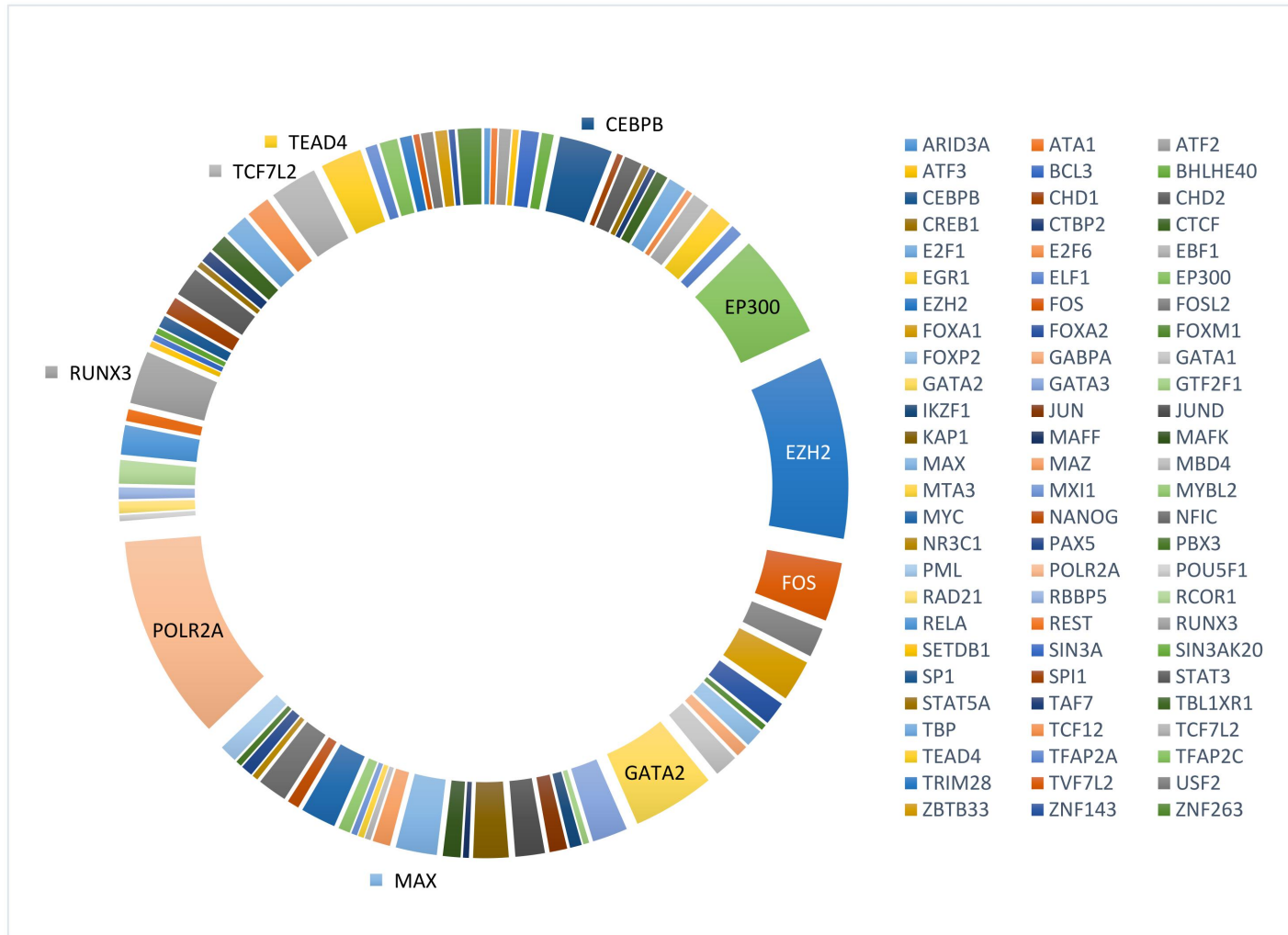

Supplement: Supplementary file 6 — Figure S6. Transcription factor binding sites subject to differential DNA methylation among 168 CpGs. Analysis of 168 differentially methylated CpGs between atrial and ventricular heart tissue revealed an overlap of 54% (90/168) of CpG loci being associated to TFBSs in various cell lines from ChIP-seq experiments performed by ENCODE (Factorbook Motifs [32]). The ten most common TFs showing TFBSs among the 168 CpGs are labelled: POLR2A, EZH2, EP300, GATA2, FOS, RUNX3, CEBPB, TCF7L2, MAX and TEAD4. Only TFBSs with Cluster Scores (out of 1000) > 300 are listed (Additional file 15: Table S2). (PDF 354 kb) [file 13148_2019_679_MOESM6_ESM.pdf]

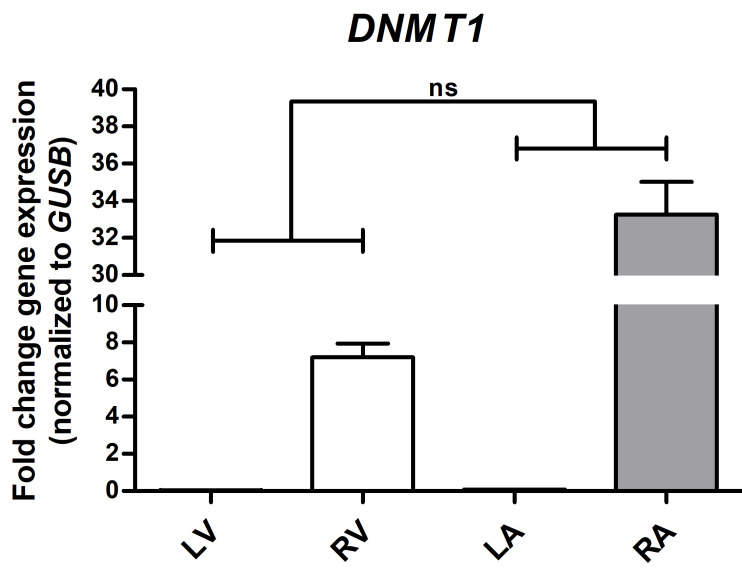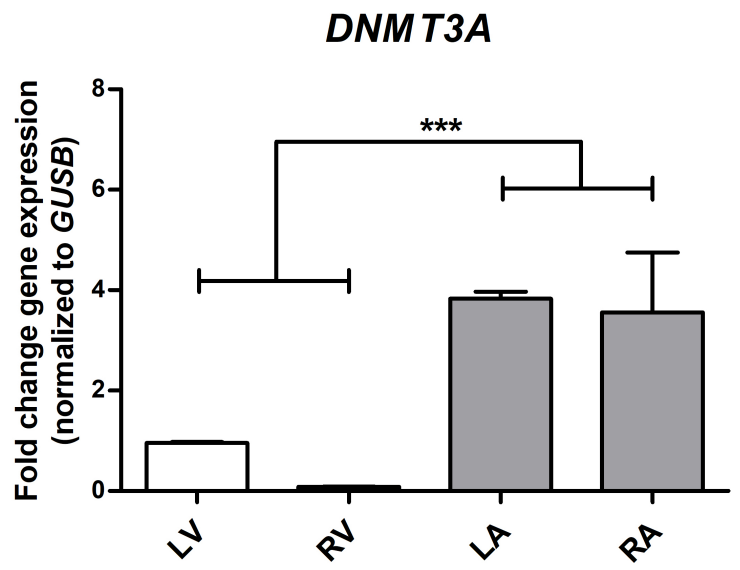

Supplement: Supplementary file 7 — Figure S7. mRNA expression of DNA methyltransferase genes in atrial and ventricular tissues. qPCR experiments were performed on atrial (LA, RA; grey bars) and ventricular (LV, RV; white bars) tissues of one patient (0126) with LV-Aneurysm. Triplicates of cDNA samples were analysed. Expression data was normalized to GUSB housekeeping gene and compared to atrial or ventricular expression (∆∆ CT method), p < 0.05 (Student’s t test), bars show mean with SEM. (PDF 547 kb) [file 13148_2019_679_MOESM7_ESM.pdf]

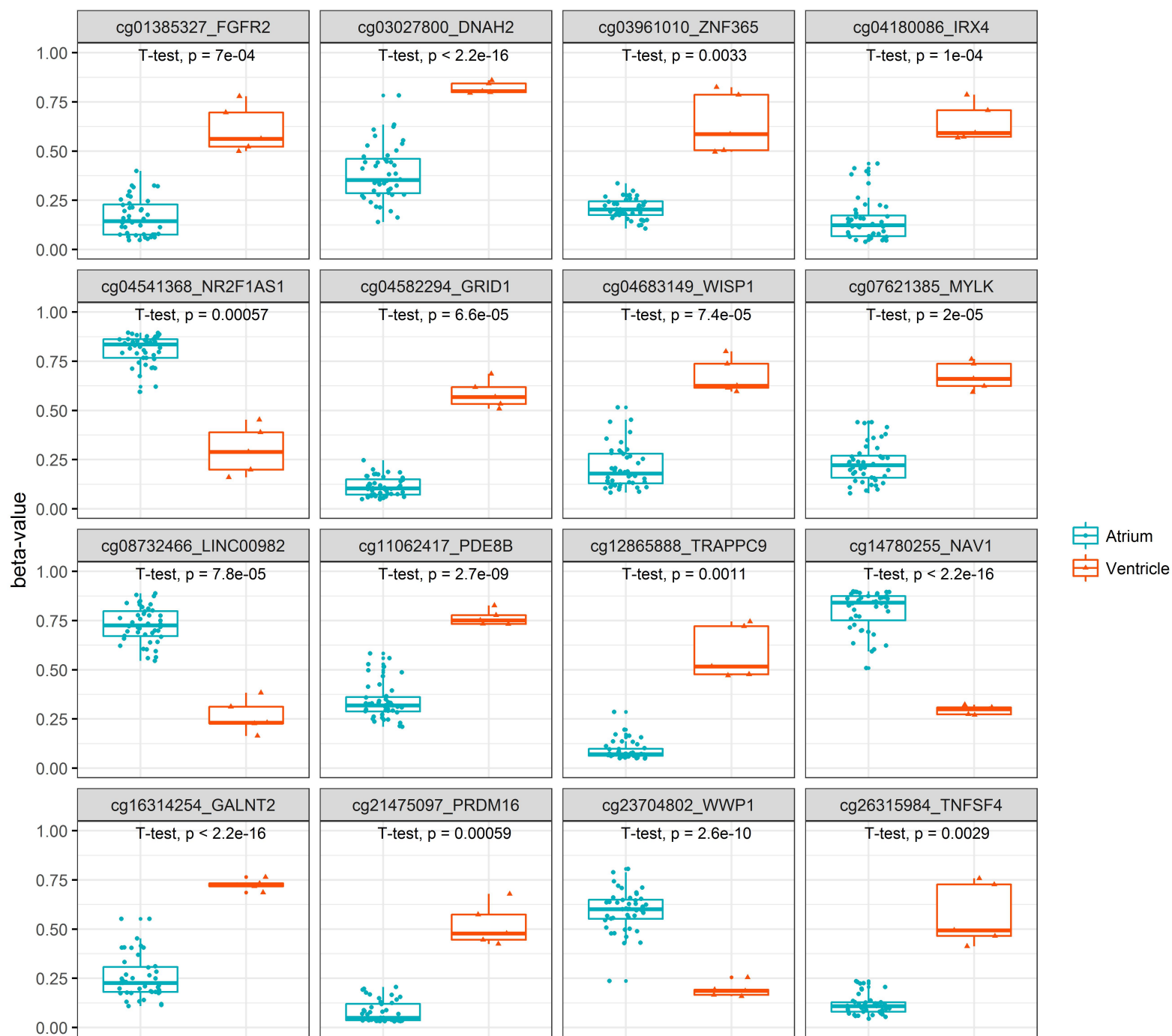

Supplement: Supplementary file 8 — Figure S8. Boxplots (t test) of 16 candidate CpG loci with differential atrial-ventricular methylation pattern. Differential DNA methylation (β-value) of 16 candidate CpG loci in atrial (n = 44) and ventricular (n = 5) cardiac tissue samples being either hypomethylated or hypermethylated in atrial tissue (coloured blue) compared to ventricular tissue (coloured red). Highly significant DNA methylation differences (p ≤ 0.0001) in atrial vs. ventricular tissues have been detected at 13 of 16 CpG loci (Welch Two Sample t test) with 9 loci displaying p values <7.76 × 10^−7^ (p values Additional file 19: Table S6). Data is presented as standard box-and-whiskers plots (whiskers, 5th–95th percentile). (PDF 3430 kb) [file 13148_2019_679_MOESM8_ESM.pdf]

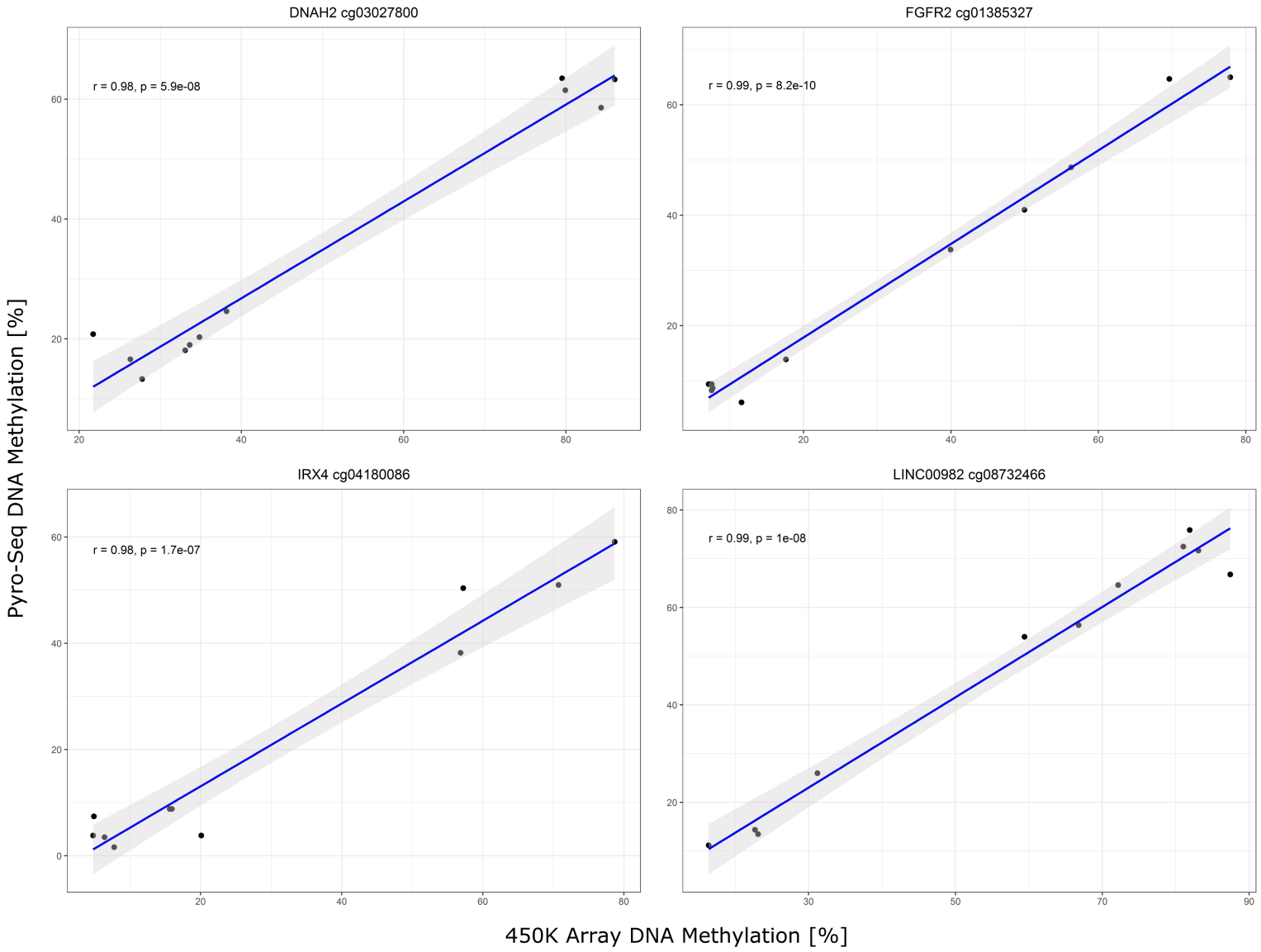

Supplement: Supplementary file 9 — Figure S9. Correlation between methylation values of 450K array and bisulfite pyrosequencing (DNAH2, FGFR2, IRX4 and LINC00982). Linear regression analysis of methylation values (percentage) at four candidate CpG loci (on DNAH2, FGFR2, IRX4 and LINC00982) from 11 cardiac tissue samples that have been subjected to 450K array analysis (β-values as percentage on x-axis) and bisulfite pyrosequencing (y-axis). The 11 samples of the verification set are listed in Additional file 14: Table S1. At all four candidate CpG loci a high correlation of the methylation values (R2 ranging from 0.98 to 0.99, and p values ranging from p = 1.7 × 10^−7^ to p = 8.2 × 10^−10^) from the two platforms, 450K array and bisulfite pyrosequencing, could be verified. (PDF 1201 kb) [file 13148_2019_679_MOESM9_ESM.pdf]

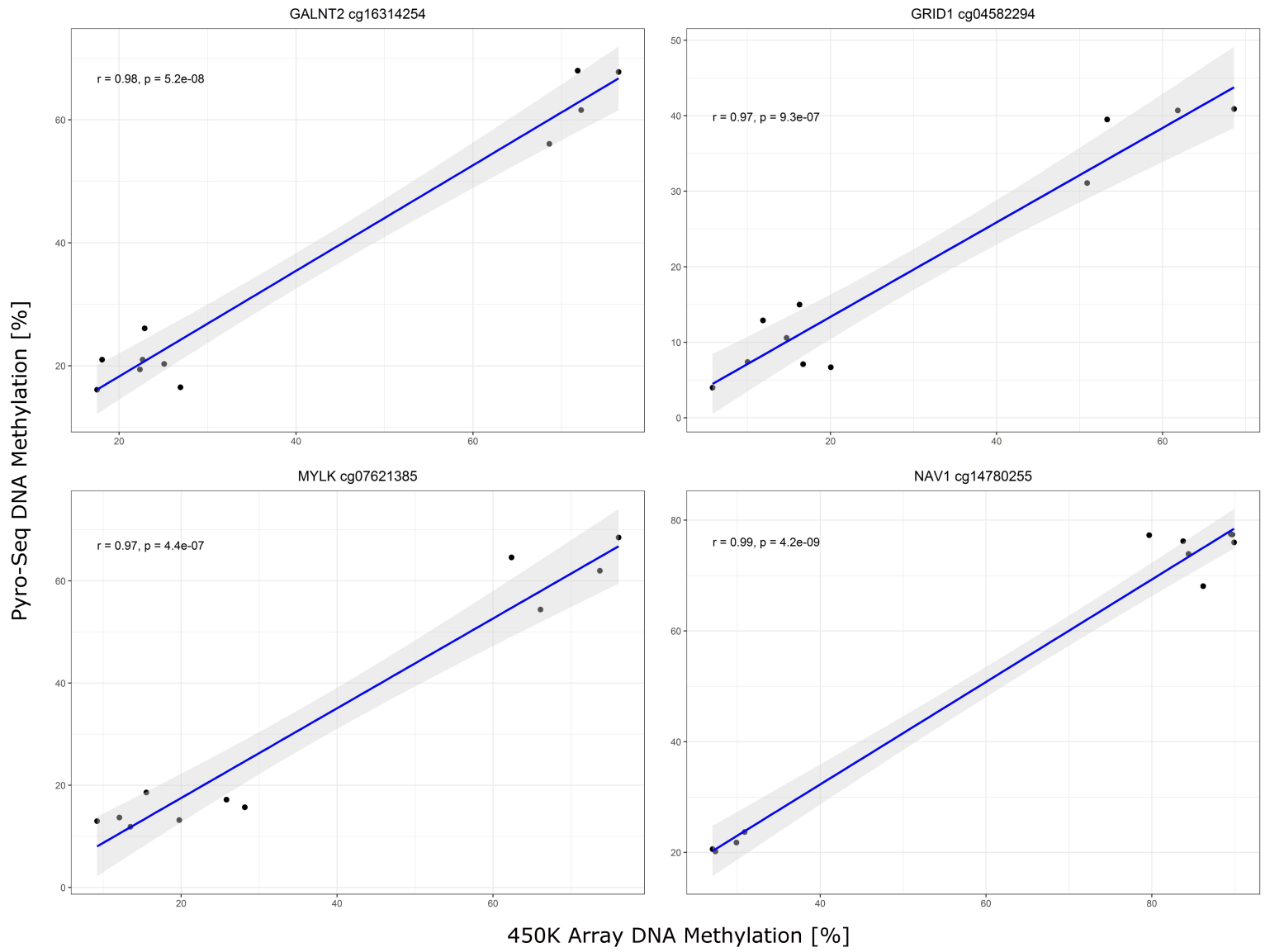

Supplement: Supplementary file 10 — Figure S10. Correlation between methylation values of 450K array and bisulfite pyrosequencing (GALNT2, GRID1, MYLK and NAV1). Linear regression analysis of methylation values (percentage) at four candidate CpG loci (on GALNT2, GRID1, MYLK and NAV1) from 11 cardiac tissue samples that have been subjected to 450K array analysis (β-values as percentage on x-axis) and bisulfite pyrosequencing (y-axis). The 11 samples of the verification set are listed in Additional file 14: Table S1. At all four candidate CpG loci a high correlation of the methylation values (R2 ranging from 0.97 to 0.99, and p values ranging from p = 9.3 × 10^−7^ to p = 4.2 × 10^−9^) from the two platforms, 450K array and bisulfite pyrosequencing, could be verified. (PDF 1143 kb) [file 13148_2019_679_MOESM10_ESM.pdf]

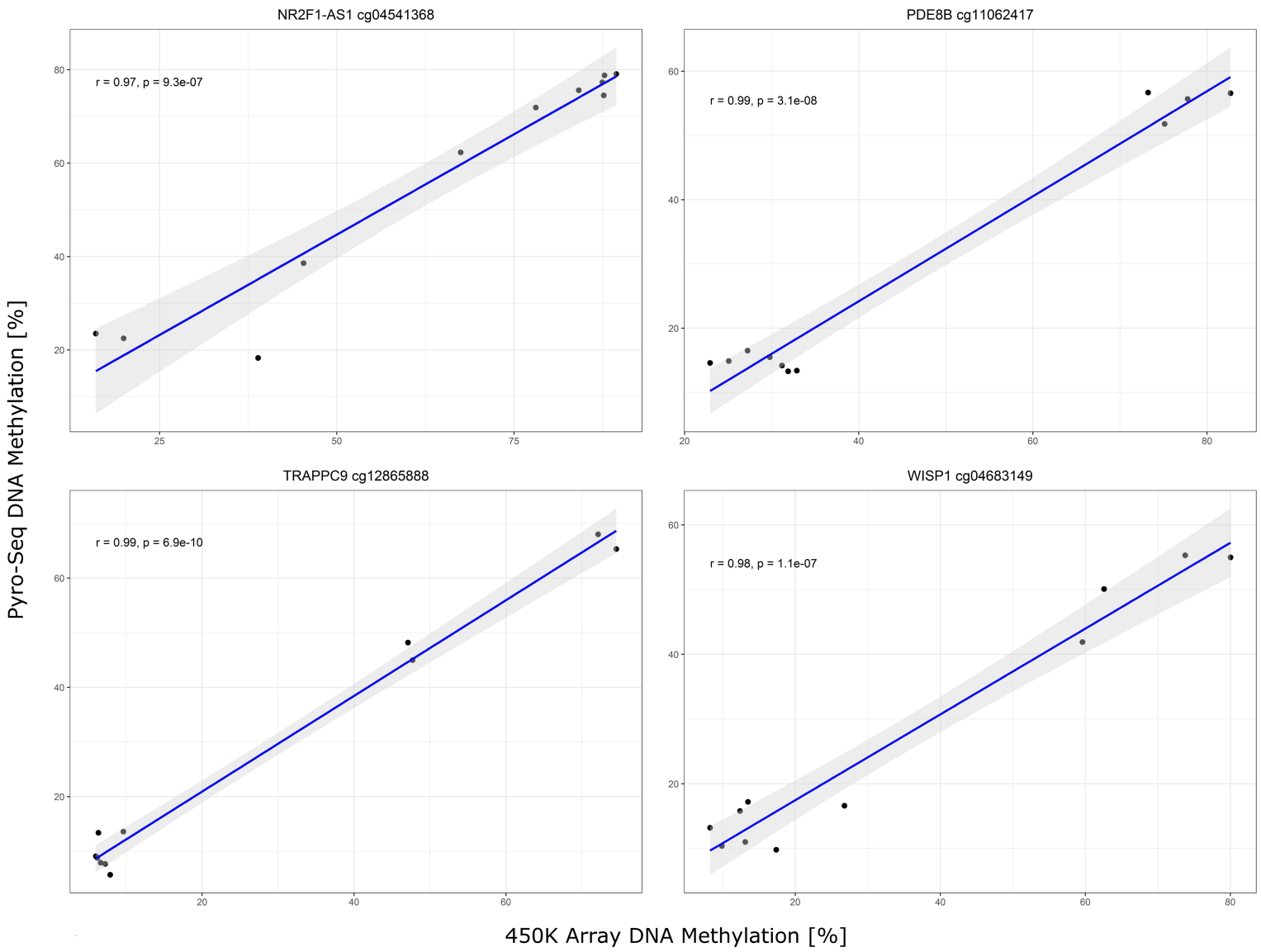

Supplement: Supplementary file 11 — Figure S11. Correlation between methylation values of 450K array and bisulfite pyrosequencing (NR2F1-AS1, PDE8B, TRAPPC9 and WISP1). Linear regression analysis of methylation values (percentage) at four candidate CpG loci (on NR2F1-AS1, PDE8B, TRAPPC9 and WISP1) from 11 cardiac tissue samples that have been subjected to 450K array analysis (β-values as percentage on x-axis) and bisulfite pyrosequencing (y-axis). The 11 samples of the verification set are listed in Additional file 14: Table S1. At all four candidate CpG loci a high correlation of the methylation values (R2 ranging from 0.97 to 0.99, and p values ranging from p = 9.3 × 10^−7^ to p = 6.9 × 10^−10^) from the two platforms, 450K array and bisulfite pyrosequencing, could be verified. (PDF 1184 kb) [file 13148_2019_679_MOESM11_ESM.pdf]

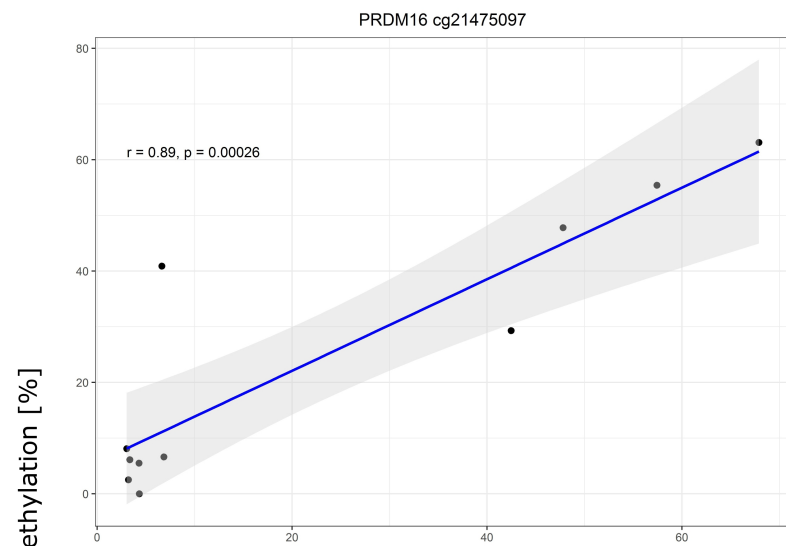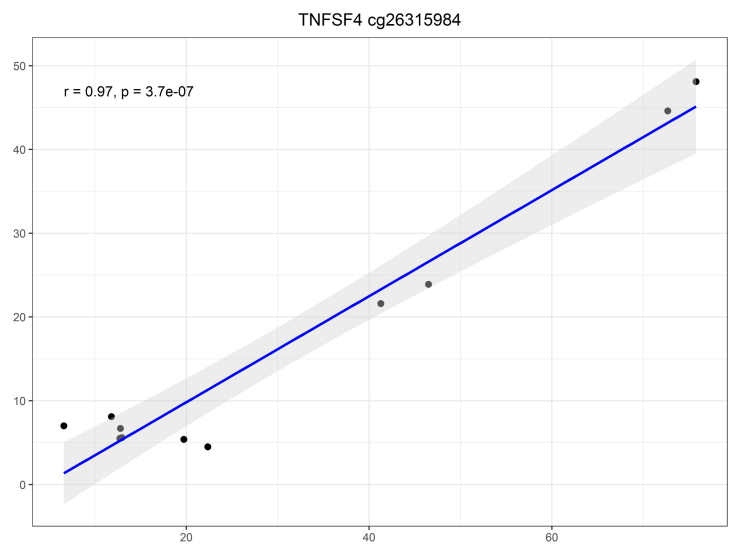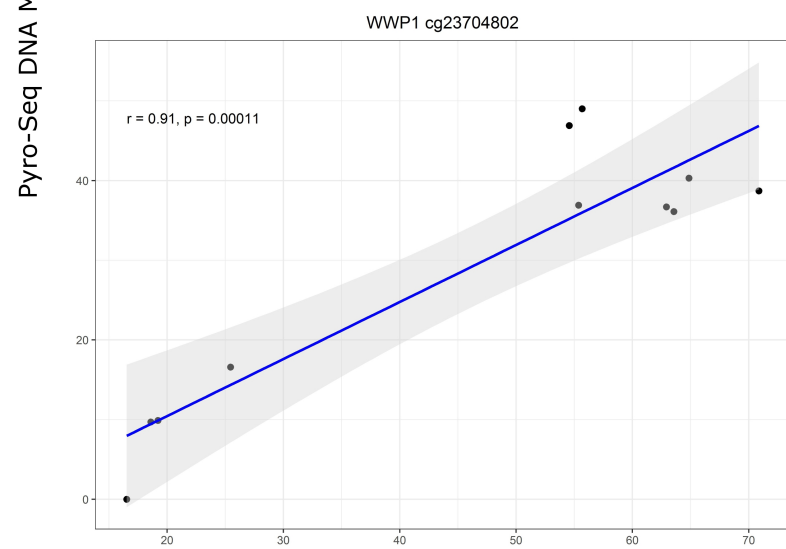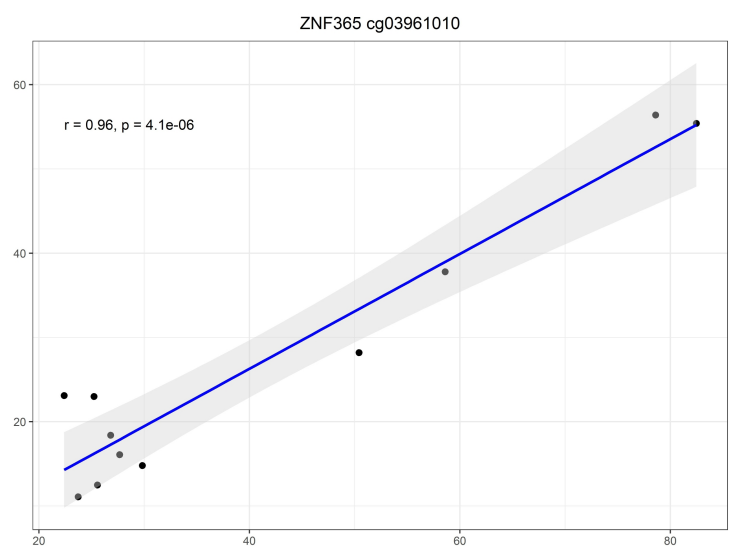

450K Array DNA Methylation [%]

Supplement: Supplementary file 12 — Figure S12. Correlation between methylation values of 450K array and bisulfite pyrosequencing (PRDM16, TNFSF4, WWP1 and ZNF365). Linear regression analysis of methylation values (percentage) at four candidate CpG loci (on PRDM16, TNFSF4, WWP1 and ZNF365) from 11 cardiac tissue samples that have been subjected to 450K array analysis (β-values as percentage on x-axis) and bisulfite pyrosequencing (y-axis). The 11 samples of the verification set are listed in Additional file 14: Table S1. At all four candidate CpG loci a high correlation of the methylation values (R2 ranging from 0.91 to 0.97, and p values ranging from p = 0.00026 to p = 3.7 × 10^−7^) from the two platforms, 450K array and bisulfite pyrosequencing, could be verified. (PDF 1164 kb) [file 13148_2019_679_MOESM12_ESM.pdf]
