# Supplementary material for: DNA methylation profiling allows for characterization of atrial and ventricular cardiac tissues and hiPSC-CMs
Source: Clin Epigenetics. 2019 Jun 11;11:89. doi: 10.1186/s13148-019-0679-0 (PMC6560887; doi:10.1186/s13148-019-0679-0)
Supplement: Supplementary file 3 — Figure S3. β-value correlation of cardiac tissue biological replicates. Linear regression analysis of β-values (448,814 loci per sample) from biological replicates (LA, RA, LV and RV; each triplicates or duplicates as shown in table) from CHD patients 0117 and 0126. All replicates showed high correlation of β-values (R2 > 0.99, p < 2.2 × 10^−16^). Scatter plots of two replicate combinations, RA1_0117-RA2_0117 and LV1_0126-LV2_0126, are depicted. (PDF 5245 kb) [file 13148_2019_679_MOESM3_ESM.pdf]

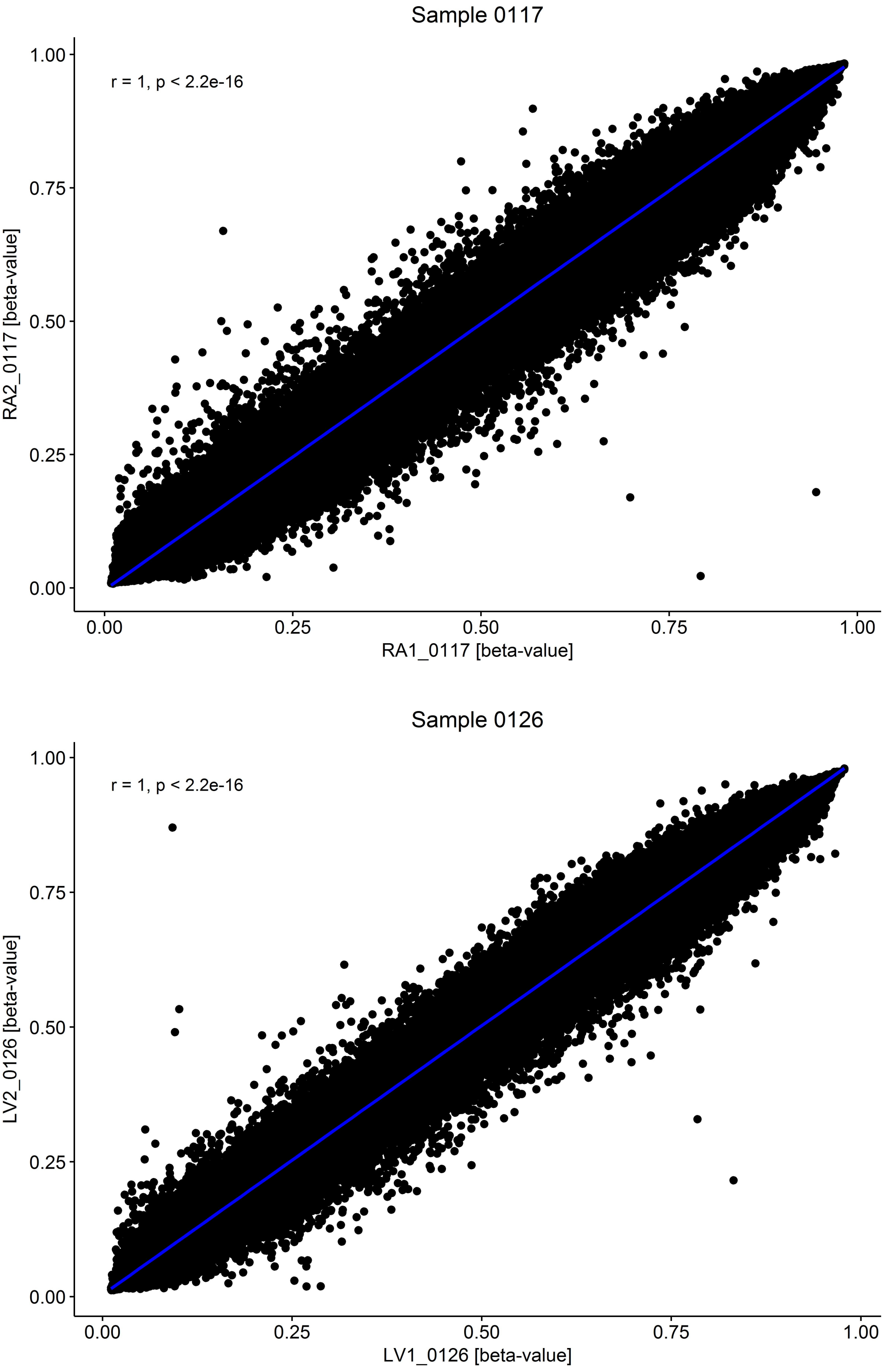

Sample ID	Regression Analysis [tissue x vs. tissue y]	R^2-value
0117	LV1 - LV2	0.9970855
	LV1 - LV3	0.9959226
	LV2 - LV3	0.9972144
	RV1 - RV2	0.9961868
	RV1 - RV3	0.9969996
	RV2 - RV3	0.9950241
	LA1 - LA2	0.9908811
	LA1 - LA3	0.9941115
	LA2 - LA3	0.997572
	RA1 - RA2	0.9971142
	RA1 - RA3	0.9970153
	RA2 - RA3	0.9985258
0126	LV1 - LV2	0.9977693
	LV1 - LV3	0.9972465
	LV2 - LV3	0.9975448
	RV1 - RV2	0.9962584
	LA1 - LA2	0.9975677
	LA1 - LA3	0.9976382
	LA2 - LA3	0.9977372
	RA1 - RA2	0.9977619
	RA1 - RA3	0.9974826
	RA2 - RA3	0.9978541
