# Supplementary material for: DNA methylation profiling allows for characterization of atrial and ventricular cardiac tissues and hiPSC-CMs
Source: Clin Epigenetics. 2019 Jun 11;11:89. doi: 10.1186/s13148-019-0679-0 (PMC6560887; doi:10.1186/s13148-019-0679-0)
Supplement: Supplementary file 13 — Figure S13. Bisulfite pyrosequencing analysis of candidate CpG loci: test of specificity of AVM pattern. DNA methylation patterns of different cell lines (grey bars, each single samples)—endothelial cells of the hiPSC line (hiPSC-EC), valve interstitial cardiac cell line (VIC, Innoprot P10462) and a non-cardiac cell line MCF7 (breast cancer cell line)—were investigated using bisulfite pyrosequencing technique to compare to the AVM patterns of cardiac tissues from patients with CHDs (discovery set, 450K array data of atrial (n = 44) and ventricular (n = 5) tissues; white bars) and in vitro cultivated atrial and ventricular subtypes of hiPSC-CMs (bisulfite pyrosequencing data, n = 4 atrial-like and n = 8 ventricular-like hiPSC-CMs; dotted bars). CpG loci with significant (p < 0.05) differences in atrial and ventricular-like hiPSC-CMs showing similar AVM pattern as compared to the pattern in primary human atrial and ventricular cardiac tissues (discovery set) are marked with ‘AVM’. ‘Tendency loci’ with similar AVM pattern, that did not reach significance are marked with ‘AVM*’ and loci showing opposite AVM pattern are marked with ‘inv’. hiPSC-ECs mostly showed lower DNA methylation values than atrial or ventricular hiPSC-CMs, human cardiac VICs revealed very low DNA methylation values (median 9.4%) over all 16 CpG loci and MCF7 cell line showed high DNA methylation values (median 77.6%). All three cell lines—hiPSC-EC, VIC, MCF—did not resemble the AVM pattern over the 16 candidate CpG loci. Data is presented as standard bar plots (mean with SEM). (PDF 6347 kb) [file 13148_2019_679_MOESM13_ESM.pdf]

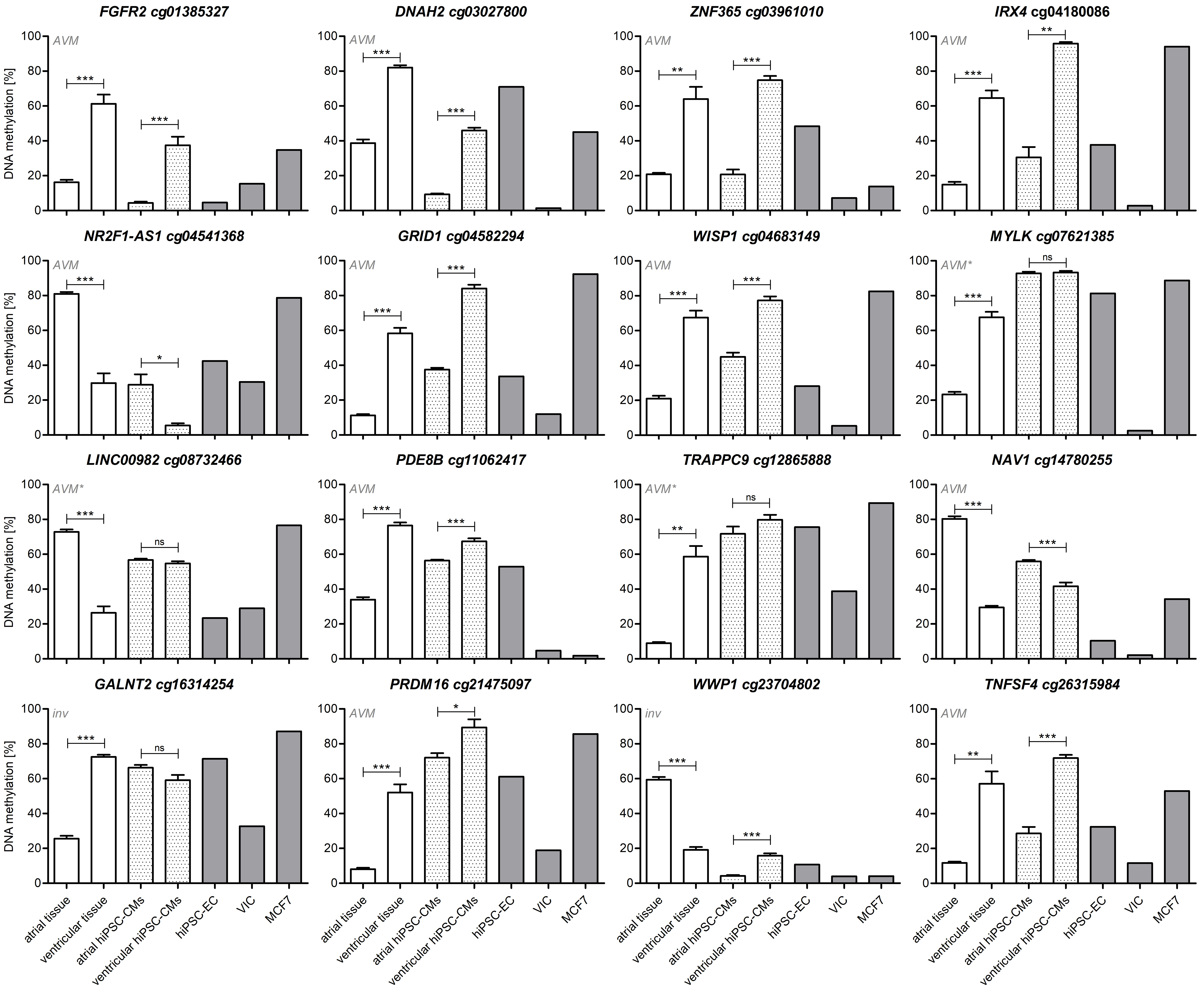
FGFR2 cg01385327
DNAH2 cg03027800
ZNF365 cg03961010
IRX4 cg04180086
NR2F1-AS1 cg04541368
GRID1 cg04582294
WISP1 cg04683149
MYLK cg07621385
LINC00982 cg08732466
PDE8B cg11062417
TRAPPC9 cg12865888
NAV1 cg14780255
GALNT2 cg16314254
PRDM16 cg21475097
WWP1 cg23704802
TNFSF4 cg26315984
AVM
AVM*
inv
***
**
*
ns
DNA methylation [%]
0
20
40
60
80
100
atrial tissue
ventricular tissue
atrial hiPSC-CMs
ventricular hiPSC-CMs
hiPSC-EC
VIC
MCF7
